# Predicting subjective ratings of affect and comprehensibility with text features: a reader response study of narrative poetry

**DOI:** 10.3389/fpsyg.2024.1431764

**Published:** 2024-10-08

**Authors:** Mesian Tilmatine, Jana Lüdtke, Arthur M. Jacobs

**Affiliations:** ^1^Department of Experimental and Neurocognitive Psychology, Freie Universität Berlin, Berlin, Germany; ^2^Centre for Language Studies, Department of Language and Communication, Faculty of Arts, Radboud University, Nijmegen, Netherlands; ^3^Donders Centre for Cognition, Department of Artificial Intelligence, Faculty of Social Sciences, Radboud University, Nijmegen, Netherlands; ^4^Center for Cognitive Neuroscience Berlin, Department of Education and Psychology, Free University of Berlin, Berlin, Germany

**Keywords:** reading, narrative cognition, empirical study of literature, aesthetic perception, rating prediction, quantitative text analysis, naturalistic stimuli

## Abstract

Literary reading is an interactive process between a reader and a text that depends on a balance between cognitive effort and emotional rewards. By studying both the crucial features of the text and of the subjective reader reception, a better understanding of this interactive process can be reached. In the present study, subjects (*N*=31) read and rated a work of narrative fiction that was written in a poetic style, thereby offering the readers two pathways to cognitive rewards: Aesthetic appreciation and narrative immersion. Using purely text-based quantitative descriptors, we were able to independently and accurately predict the subjective ratings in the dimensions comprehensibility, valence, arousal, and liking across roughly 140 pages of naturalistic text. The specific text features that were most important in predicting each rating dimension are discussed in detail. In addition, the implications of the findings are discussed more generally in the context of existing models of literary processing and future research avenues for empirical literary studies.

## Introduction

Reading is a complex process. The meaning of individual words has to be decoded and integrated into a larger understanding of clauses, sentences, or entire paragraphs. In the case of fiction reading, this larger understanding of sentences and paragraphs again has to fit into the understanding of an overarching narrative. On top of that, narrative fiction typically entails considerably more literary language than non-fiction texts. Stylistic deviations from the linguistic norm are used to create non-familiar perspectives during reading, which is also known as *foregrounding* (Van Peer et al., 2007; Van Peer et al., 2021). For example, suddenly changing the rhyme scheme for dramatic effect would be stylistic foregrounding, and describing a desert as an *endless sea of sand* would be a sort of semantic foregrounding. Narrative and foregrounding aspects of literary texts both enforce active engagement of the reader. The text does not only convey factual information, but also evokes feelings as a response to both the narrated story and the style of language (Jacobs, 2015, 2023; Mar et al., 2011; Miall and Kuiken, 1994a, 1994b). In other words, literary reading is complex, but can be rewarding, in both cognitive and emotional terms.

**Figure 1:**
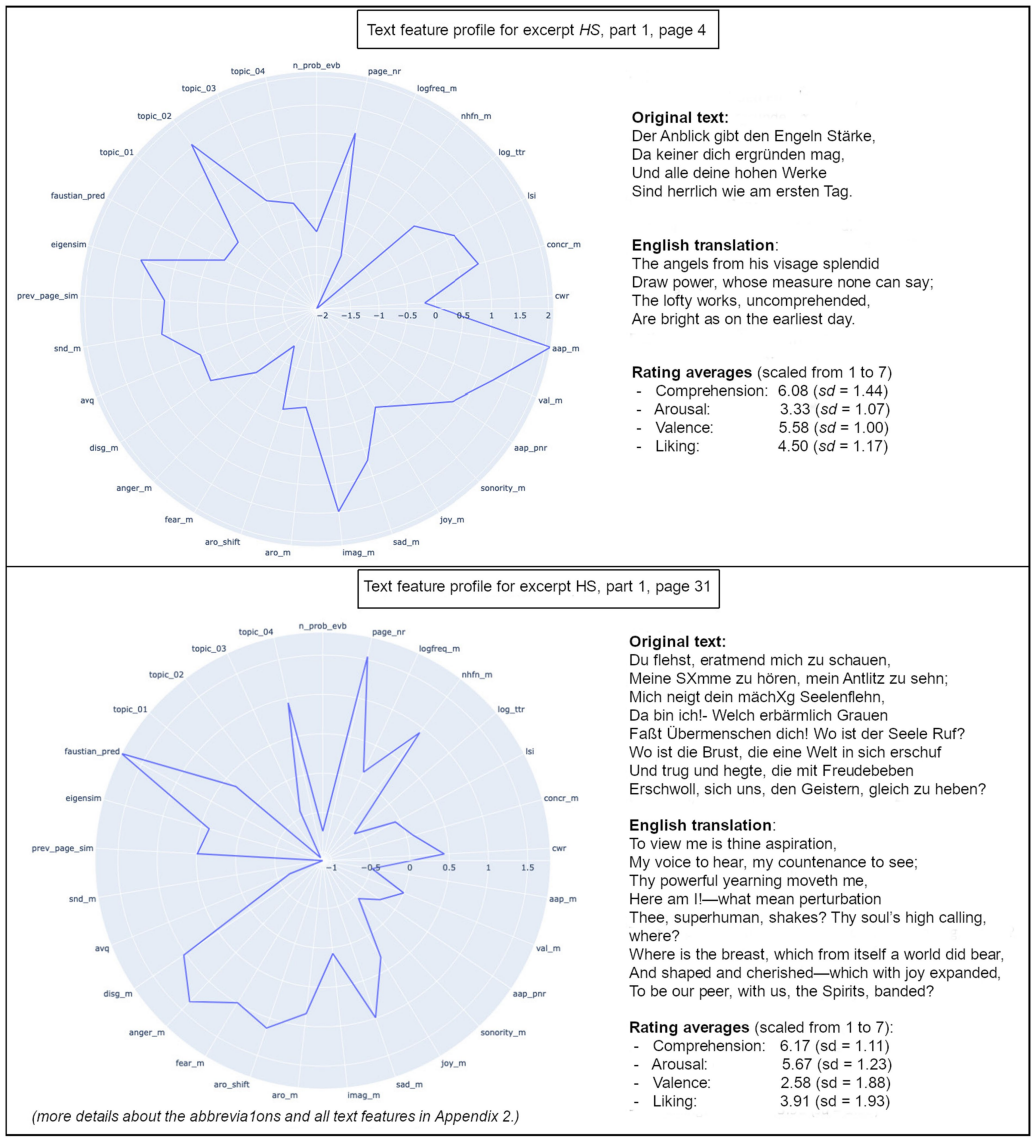
Text profiles of two selected pages (translations in original meter by Bayard Taylor). More details about the abbreviations and all text features in Appendix 1.

Models of literary processing describe reader responses to fiction and verbal art in terms of neuronal activity, subjective experiences, and/or objective (i.e., intersubjectively examinable, directly observable) behavior. In any case, these models have to find a way to account for the complexity of the process (Jacobs, 2015, 2023; Jacobs and Willems, 2018; Willems and Jacobs, 2016). This is true for studies testing such models as well, in the sense that the stimulus material should ideally contain the potential to elicit a wide variety of possible responses from the readers. The longer a literary text is, the more it can be expected to naturally vary in properties that potentially elicit specific reactions in readers. For this paper, we chose a work of *narrative poetry*. That is, a work of literary fiction that tells a relatively long narrative using highly literary stylistics as stimulus material, thereby containing typical characteristics for narrative as well es poetic texts. Then, we calculated quantitative text features to describe this work of narrative poetry on a level of relatively small text units. For each text unit, we used the resulting features to predict the emotional responses the text elicited in readers, in the form of their subjective ratings. A direct example-wise comparison between the text feature profiles of two pages can be found in Figure 1.

### Underlying processing mechanisms for literary texts

The *Neurocognitive Poetics Model of Literary Reading* (Schrott and Jacobs, 2011; Jacobs, 2015; Jacobs, 2023), or NCPM for short, specifies a central distinction between immersive and aesthetic processing. The former is expected to lead to fluent reading with a focus on the narrative. It should occur, for example, when reading suspenseful prose (*cf.* Hsu et al., 2014; Lehne et al., 2015; Wallentin et al., 2011). The latter is linked to slowed-down reading and appreciation of textual beauty most often associated with reading poetry (*cf.* Schrott and Jacobs, 2011; Jacobs, 2015; Jacobs, 2023, Miall and Kuiken, 1994a, 1994b).

The NCPM thus suggests two different approaches of readers emotionally processing literary texts, which are associated with differing outcomes on the subjective-experiential, neuro-cognitive, and objective-behavioral levels in the aforementioned studies. One is an immersive trajectory that heavily depends on gathering information to contextualize a story in its world, the other an aesthetic trajectory, in which the reader ponders about the emotional impact of the way in which language is used in the text. Previous studies have investigated either prosaic narratives (e.g., Lehne et al., 2015; Altmann et al., 2012; Eekhof et al., 2021; Hartung et al., 2021) or rather short poems (e.g., Lüdtke et al., 2014; Wassiliwizky et al., 2017). According to the above framework, this would always bias one of the two theoretical trajectories to be dominant. This results in a lack of clarity about how these two trajectories could interact with one another. The present study aims to achieve more clarity about such an interaction on the subjective-experiential level.

With our stimulus material of narrative poetry, we could expect three possible scenarios in the terms of the NCPM: Dominant immersive processing, dominant aesthetic processing, or a hybrid sort of processing that somehow incorporates elements of both immersive and aesthetic processing. In principle, there is also a fourth scenario in which neither immersive nor aesthetic processing takes place in any form. However, this would imply a complete failure of literary processing for narrative poetry. Literary processing can certainly fail for individual readers (not) engaging with individual texts, and the reasons for that are as multifaceted as human language processing is in general (Graf and Landwehr, 2015; Kidd et al., 2018; Tilmatine et al., 2021; Mak et al., 2022). Given the existence of various works of narrative poetry which receive exceptional critical acclaim, it seems highly unlikely that this fourth scenario would be generally true for established works of literature. Nonetheless, textual comprehensibility must be accounted for in studies like this one, especially as comprehensibility is also tied to liking, albeit in complicated ways (Güçlütürk et al., 2016).

### The emotional aspect of literary processing

According to the *Panksepp-Jakobson hypothesis –* verified in several studies (Ziegler et al., 2018) –, humans use the same mechanisms for emotional processing in fiction processing as in real life (Jacobs, 2015). Therefore, the two different trajectories of the NCPM both contributing to emotional reader responses must translate into two different aspects of classic emotional processing. And duality is common in emotion theory: Most dimensional approaches to understanding emotion know some variations of concepts that are similar to the continuous duality of *valence* versus *arousal* (Rubin and Talarico, 2009).

While arousal has been related to narrative immersion, for example in the form of suspense (Kaakinen and Simola, 2020), the aesthetic trajectory has been linked to valence (Jacobs and Kinder, 2019). The link between appreciation of literary beauty and valence is not a simple linear one though; for instance, certain studies suggest a correlation between poetry appreciation and both positive and negative valence (Jacobs et al., 2016; Kraxenberger and Menninghaus, 2017). It thus seems like strong valence, positive or negative (as opposed to neutral valence), is one of the better quantitative predictors of aesthetic processing potential. A stronger role than valence, however, May be played by arousal-related social cognition processes like identification and suspense, as well as the *reading flow*, which is related to comprehensibility (Altmann et al., 2012; Thissen et al., 2018). As mentioned before, comprehensibility in itself is an important element in predicting both NCPM trajectories. This is because a lack of understanding May negatively impact the emotional response elicited by both prosaic narratives (Eekhof et al., 2021; Thissen et al., 2018) and poetic foregrounding (Harash, 2022; Scapin et al., 2023).

In sum, there are reasons to assume that arousal ratings are an indicator of the activation of the immersive processing trajectory, whereas valence ratings should relate more to the aesthetic processing trajectory. We have also established that both of these assumptions are only true if comprehensibility is accounted for, as well.

### Text-based prediction of the average reader response to literature

A lot of predictive potential for reader responses is extractable from the text itself, in the textual cues that the author left for the readers to pick up. Authors can be identified based on their usage of language in song poetry (Mendhakar and Tilmatine, 2023) and authorial sentiment for expressively opinionated texts can be extracted fairly successfully with the assistance of deep-learning tools nowadays (Radford et al., 2017; Zhang et al., 2018; Jacobs and Kinder, 2020). Specifically multivariate sentiment analyses have been fairly successful in the endeavor to quantify more generalized emotional loadings in texts (Kim and Klinger, 2019; Jacobs et al., 2020), which can be linked to the concept of a linguistic *emotion potential* (Winko, 2023; Schwarz-Friesel, 2015). In such multivariate sentiment analyses, a wealth of text features that are computed by tools like *Séance* (Crossley et al., 2017) or *SentiArt* (Jacobs and Kinder, 2019) based on either individual words, bi-and trigrams or entire sentences have been shown to serve as fairly accurate predictors for the response of readers to both prose and poetry (for a recent comprehensive review see Jacobs, 2023).

For example, there is evidence that both surface and affective semantic features of the text predict eye movements in short sonnets (Xue et al., 2020; Xue et al., 2023) and that emotionally evocative text features predict affective ratings in short poems (Ullrich et al., 2017; Hugentobler and Lüdtke, 2021), sonnets, song lyrics, or entire books (Jacobs et al., 2020; Jacobs, 2023). These predictions are cross-validated by the fact that standard corpora of valence ratings for single words are similarly well predictable by those tools (e.g., Bestgen, 1994; Jacobs and Kinder, 2019; Lüdtke and Hugentobler, 2022). For short prosaic texts (excerpts and short stories), text features like word valence have been found to predict suspense and immersion ratings, as well as the neural correlates of empathy, mental simulation, predictive inference, and fear (Hsu et al., 2014; Lehne et al., 2015; Mak et al., 2023).

Computational text-analysis tools including multivariate sentiment analysis have thus been shown to predict emotional reader responses pretty well on the level of single words, poems, short prosaic texts, or whole books. They can be considered an excellent way to understand the interaction between readers and literary texts. On the other side of the interaction, we have discussed that four aspects of the reading experience May together cover a reasonable portion of the emotional reader response, namely valence, arousal, comprehensibility, and liking. These four aspects can be linked to the two processing trajectories of the NCPM: The immersive trajectory should lead to fluent reading, which is associated with comprehensibility and arousal. Meanwhile, the aesthetic trajectory should lead to dysfluent reading, which in turn is associated with valence and liking (Schrott and Jacobs, 2011; Jacobs, 2015; Jacobs, 2023). In that light, two main questions for this study emerge. First, do subjective ratings of valence, arousal, comprehensibility and liking show the differences and intercorrelations that we expect on the basis of the emotional reader response predictions of the NCPM? Second, can these ratings be predicted by the text features associated with both processing trajectories? And if so, which specific text properties play which role in the prediction of which rating dimension?

In this respect, we hypothesized that the ratings of comprehensibility would correlate with the ones for liking, because understanding a narrative should be a pre-requisite for enjoying its content. Possibly, the relationship between liking and comprehensibility ratings could also follow the inversed U-shape sometimes argued for narrative texts. If a quadratic fit performs better than a linear fit, this could be an indication that readers prefer narrative texts that are neither too complex nor too simplistic in their writing style (Berlyne, 1970; Errington et al., 2022; Jacobs, 2023). In a similar vein, we expected the arousal ratings to correlate with both the comprehensibility and liking ratings, as they are our chosen window into the immersive processing trajectory. Narrative comprehensibility is probably less important for the aesthetic processing trajectory of the poetic elements in the text. For that reason, we expected valence ratings to be less correlated with comprehensibility ratings, but as an element of appreciation to still be correlated with liking ratings. As arousal and valence are often conceptualized as two independent dimensions in the duality of emotion, we expected their respective ratings to be less correlated (Russell and Barrett, 1999). Experimental evidence has shown the arousal-valence ratings relation follows a U-shape, i.e., both positive and negative valence being related to high arousal (Lang, 1994; Jacobs et al., 2015; Kron et al., 2015).

We then selected a set of 29 text features that we expected to be important predictors for all or some of the four rating dimensions (Appendix 1), most of which were inspired by a previous study on profiling the comprehensibility, emotional tone, and topics of longer texts (Jacobs and Kinder, 2021; Jacobs, 2023). Specifically, we hoped to find four independent sets of features that each uniquely predicts one rating dimension. To account for valence and arousal ratings (*cf.* Ullrich et al., 2017), we selected vector-based features that measure the average emotional load of the semantic field of content words in a text unit. Specifically, we expected *aesthetic-affective potential* (AAP) from *SentiArt* (Jacobs and Kinder, 2019) to be the most important predictor for valence ratings, and the *arousal potential* from GLEAN (Lüdtke and Hugentobler, 2022) to be the most important predictor for arousal ratings. To specifically predict comprehensibility ratings, we included surface text features like the *logarithmic type-token ratio*, a measure of the morphological complexity of a text unit (Kettunen, 2014), and higher-level ease-of-processing measures like the average *semantic neighborhood density* (Fieder et al., 2019; Hameau et al., 2019; Jacobs, 2023), a measure calculated with vector-based deep learning for each content word. The text predictor we expected to be most important for comprehensibility ratings was the page average of *logarithmic word frequency* (Crossley et al., 2011; Smith and Levy, 2013; Xue et al., 2020).

Finally, we also included text features that we thought to be directly linked to the liking of a continuous text. Basic emotions are the driving forces behind high-level stimulus appreciation (Simonton, 1990; Delmonte, 2016; Jacobs et al., 2016). As we did not know whether narrative poetry processing is dominated by the aesthetic or the immersive trajectory, we included text features related to both. The AAP includes both elements of emotional valence and of verbal beauty (Jacobs and Kinder, 2020; Jacobs, 2023), so it was a strong contender to be the most important predictor of liking ratings, as well as an indicator of the role of the aesthetic processing trajectory for liking ratings. Regarding the immersive trajectory, we expected the strongest emotion-related predictor to be the arousal potential. Next to that, we also included text features more related to the narrative itself, such as measures of the probability of narrative event boundary occurrences (*cf.* Radvansky and Zacks, 2017; Geerligs et al., 2021) or the presence of the text’s global topics in a text unit, which May contribute to individual liking patterns of narratives (Mak et al., 2022).

We first ran the analyses with the full set of 29 text features predicting the four rating dimensions per page. Based on that, we created reduced models with the seven best-performing text features for each rating dimension. Based on these reduced models, we created bagged predictions for each rating dimension (Breiman, 1996; Hastie et al., 2009). Finally, we also compared the bagged predictions with the actual ratings using moving average windows along the course of the narrative.

## Methods

### Material

Johann Wolfgang von Goethe’s *Faust – Der Tragödie Erster Teil*, written in 1808, is a long and well-respected work of rhyming poetry with an intricate narrative. We selected two excerpts of roughly equal length (565 lines for excerpt “HS,” 519 lines for “MG”), both made up of multiple chapters of the play, from a modern transcription (1986). Both excerpts together consisted of 7,077 words (2,397 without duplicate words), arranged in 1084 lines, describing 26 scenes of slightly varying lengths. We subdivided the two excerpts into pages, following three guidelines with the following priorities: (1) There should be a narrative sense to the division into pages; (2) No chain of rhymes should be interrupted; (3) No page should consist of less than 4 or more than 12 lines. This subdivision into s had two reasons. For one, practicality: This format makes it easier to replicate this study with additional online measures like eye-tracking and fMRI. It also roughly follows the segmentation format used for the long narrative in the aforementioned study by Jacobs and Kinder (2019).

The source text is written as a play with stage directions noted along the verses (e.g., “*Wagner* in a nightcap and a nightgown enters the stage with a lamp in his hand”). For this study, we did not present the subjects any of these, but only numbered verses. However, we did add in-text quotation marks whenever the speaker changed (all verses in *Faust I* are direct speech by characters), as well as occasional context between the pages – clearly marked as such – with short overviews of the scene and the speaking characters in it. The subjects were told to only rate the original verses, and we only used the original verses for the textual analysis and prediction. The entire text and context can be found in Appendix 1.

We calculated 29 quantitative text properties measuring surface, affective-semantic, syntactic, and narrative-topical text aspects to describe the emotional loadings and writing style of each page. Detailed information about them, including their calculation methods, can be found in Appendix 2.

### Participants

From the subject pool of the Freie Universität Berlin, 31 participants, all of which university students, took part in this study. The group that read excerpt HS consisted of 15 of them (13 female, 2 male, mean age: 23.73; SD = 6.11, 12 native speakers, mean completion time: 2703.67; SD = 887.08) and the group that read excerpt MG of the other 16 (12 female, 4 male, mean age: 25.75; SD = 9.54, 15 native speakers, mean completion time: 2728.00; SD = 711.25). The study was run entirely via the internet surveying tool *SoSci*.^1^ Each participant received course credits as compensation. The ethics committee of the FU’s Department of Education and Psychology approved the experimental procedures in this study (FU reference nr. 006/2021).

### Procedure

**Figure 2:**
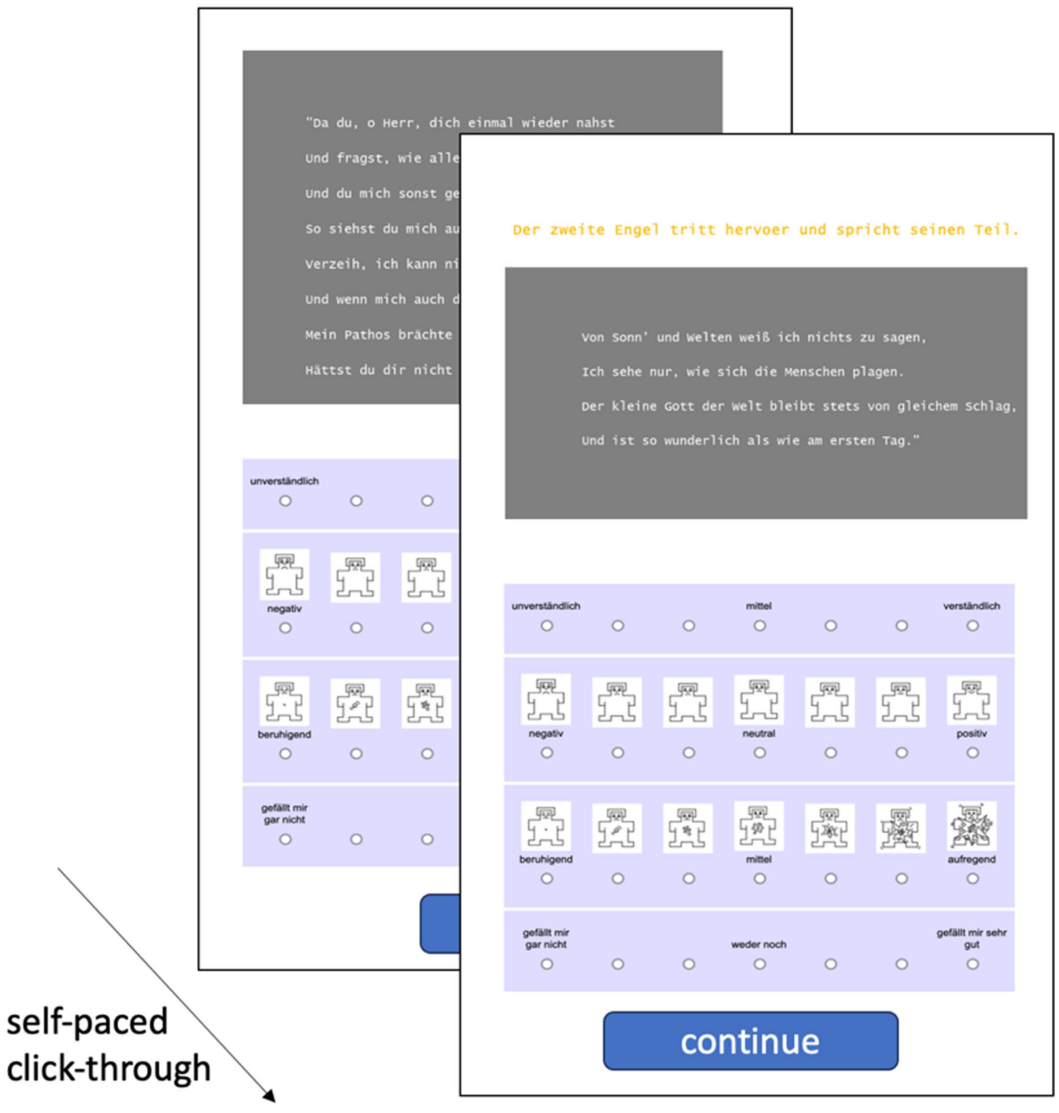
Example of a page (left) and the 7-point Likert scales used to survey subjects on their ratings for each page on Comprehensibility, Valence, Arousal, and Liking (right). In the experiment, the scales were placed beneath the text. Experimental flow chart; subjects clicked in their own pace through the pages of their excerpt and rated them one by one on the four dimensions Comprehensibility, Valence, Arousal, and Liking. Context appeared in another font color above the actual text and was clearly marked as such. The subjects were instructed to only rate the original text.

The subjects went through a self-paced reading task for one of the two excerpts. They were instructed to carefully read each page and to evaluate it on four different 7-point Likert scales measuring Comprehensibility, Valence, Arousal, and Liking. As visible in Figure 2, each scale was characterized by different labels for the extreme values as follows (freely translated from German to English): *incomprehensible* vs. *comprehensible*, *negative* vs. *positive*, *calming* vs. *exciting*, *I do not like it at all* vs. *I like it a lot*. The second and third scale were accompanied by little cartoons giving a visual support for the intensity of Valence and Arousal for each point of the scale, respectively (Bradley and Lang, 1994). Each page text slide and the four corresponding scales were shown together on the same screen, until the subject clicked to continue to the next screen in their own pace (see Figure 2).

After going through all s, the subjects filled in a survey about their reading behavior, they went through list B of the German-language version of the *Author Recognition Test* (Grolig et al., 2018), and they rated a number of pre-selected quotes of unused parts of Goethe’s Faust on familiarity. Apart from the ART, these data were mostly collected for analyses that are not part of this paper.

## Data analysis and results

First, we checked the plausibility of the ratings for each subject and excluded data accordingly. Then, we averaged each rating category (Liking, Arousal, Valence, and Comprehensibility) across the subjects on the page level. This resulted in four rating variables, each of which consisted of as many observations as there were pages (*N* = 141, see Table 1 for the descriptive statistics for the four rating variables). Then, we ran the analyses for this study in three steps. In the first step, we analyzed the rating behavior itself, specifically the inter-correlations between the four rating dimensions. As a second step, we tested the predictive accuracy of various text features for the comprehensibility, valence, arousal, and liking ratings, respectively. As a third step, we explored whether we could improve predictive accuracy by smoothing with moving average windows on the supra-page level.

**Table 1:** Descriptive Statistics for rating dimensions (7-point scale) after excluding subjects.

	Mean	Standard deviation	Range
Valence	3.89	0.83	1.94 to 6.08
Arousal	4.43	0.48	3.33 to 5.92
Liking	4.34	0.54	3.13 to 5.67
Comprehensibility	5.41	0.71	3.67 to 7.00

### Step 1: analyses of rating behavior

Here, we first checked general assumptions about the subjective ratings of text comprehensibility. Narrative poetry from the 19th century might be challenging to understand for the average contemporary reader. For that reason, we used the comprehensibility ratings to identify individual readers who May not have sufficiently understood the text to provide reliable content ratings for the other three rating dimensions. After that, we took a closer look at the page-level averages across subjects of the valence, arousal, and liking ratings, and how they interacted with each other in our data set.

#### Assumption checks about text comprehensibility

**Figure 3:**
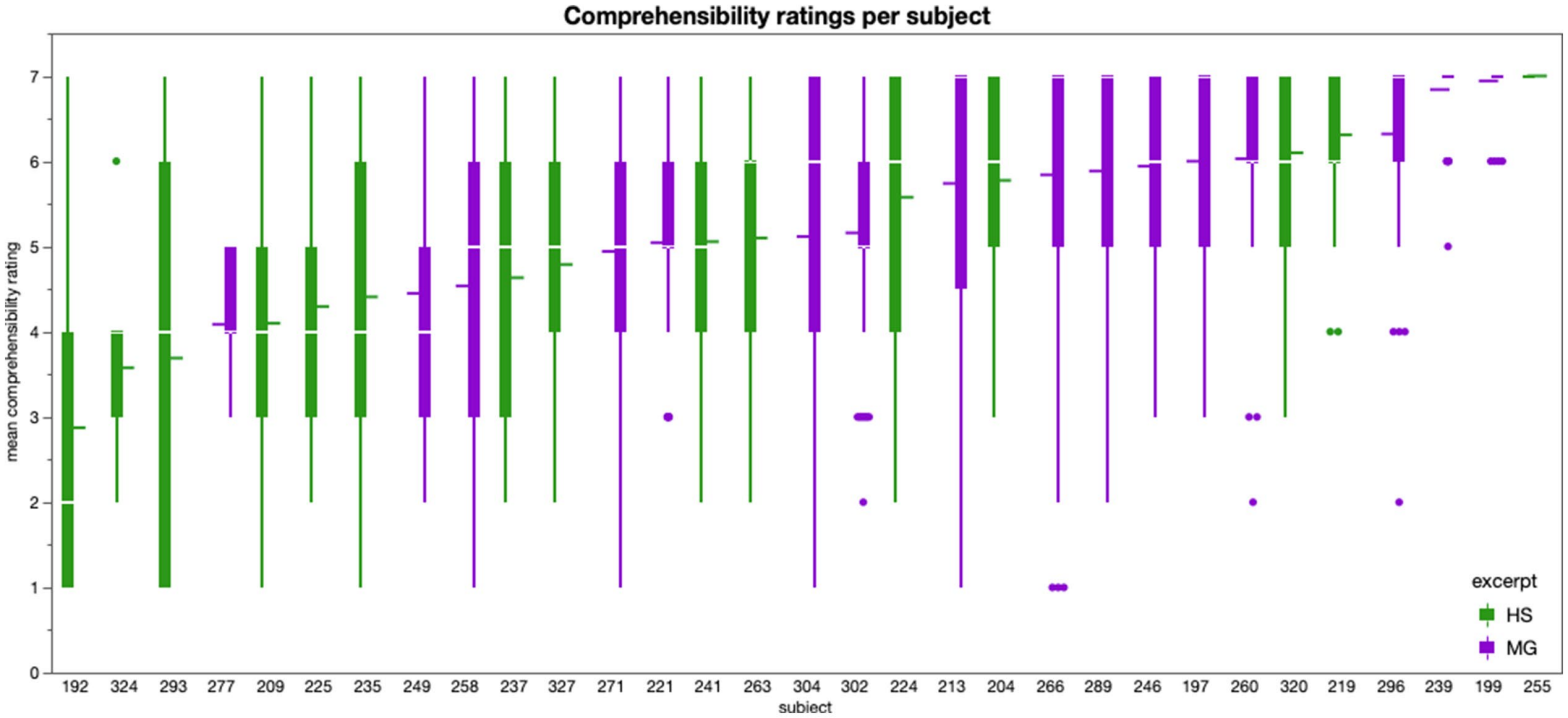
Comprehensibility ratings for each subject, boxplots represent the ratings per page (for excerpt HS in green and MG in violet); the three subjects on the very left of the graph were excluded from further analyses because they gave average Comprehensibility ratings in the lower half of the 7-point scale.

As visualized in Figure 3, there was quite some variance between subjects in terms of self-reported text comprehensibility. We removed three subjects (IDs: 192, 293, 324) from all further analyses because their ratings were on average lower than 4, the theoretical mean of the comprehensibility scale. Subject 293 also had the lowest score of all subjects on the author recognition test (34.67% correct), which can be regarded as a useful estimate of literary knowledge (Moore and Gordon, 2014). Subjects 192 (42.67% correct) and 324 (52.00% correct) were closer to, but still below the mean value of our sample for author recognition (percentage of correct answers *M* = 55.66, SD = 15.31). After removing these subjects from the dataset, we averaged the ratings of all remaining subjects per page and used these mean values for the rest of the analyses.

#### Interrelations between the subjective rating dimensions

Linear regressions (see Table 2) showed that the comprehensibility values were highly correlated with liking values, with a significant positive linear fit [*beta* = 0.565; *t* (1,138) = 5.56; *R^2^* = 0.18]. A quadratic fit between these two rating groups did not perform better [*beta_linear_* = 0.565, *beta_quadratic_* = −0.059; *t* (2,137) = 5.50; *R^2^* = 0.18], and did not show the hypothesized inverted U-shape. No significant linear fit was found for the relationship of the comprehensibility values with those of valence [*beta* = −0.016; *t* (1,138) = −0.22; *R^2^* < 0.01] and arousal [*beta* = 0.061; *t* (1,138) = 0.48; *R^2^* < 0.01]. Valence ratings were found to be a significant positive linear predictor for liking ratings [*beta* = 0.228; *t* (1,138) = 4.41; *R^2^* = 0.12], which was not the case for the relationship between the ratings of arousal and liking [*beta* = 0.109; *t* (1,138) = 1.15; *R^2^* < 0.01].

**Table 2:** Overview of relationships between rating variables (linear regressions).

	Comprehensibility	Valence	Arousal
Valence	*t*(1,138) = −0.22		
Arousal	*t*(1,138) = 0.48	*t*(1,138) = −1.41	
Liking	*t*(1,138) = 5.56*** R^2^ = 0.18	*t*(1,138) = 4.41*** R^2^ = 0.12	*t*(1,138) = 1.15

The distribution pairs marked with asterisks are significantly correlated (*p < 0.05; **p < 0.001; ***p < 0.0001).

### Step 2: text properties predicting rating behavior

For this step, we ran artificial neural networks (ANN) to predict each rating variable with specifically constructed models of seven different text features. We ran the ANNs over both excerpts together (ANN hyperparameters inspired by Xue et al., 2020: 5-fold cross-validation, one hidden layer with 10 nodes, hyperbolic tan activation function, learning rate = 0.1, number of tours = 10, number of models = 10).

Unlike other statistical approaches, this ANN approach allowed us to assess the importance of each individual predictor even though some of them might correlate or be masked behind non-linear correlations (Borsboom et al., 2021). This approach has proven to be prudent in previous similar studies (Xue et al., 2020; Musso et al., 2020; Eckstein et al., 2023). The effect size estimates were generated by the statistical software *JMP 17* (SAS Institute Inc., 1989–2023), which produces both a main effect and a total effect for each predictor in a model. The total effect includes interaction effects with other predictors and thus gives a more complete insight into a predictor’s contribution to a model effect.

We always started with the same full model of 29 features as predictors, then reduced the model to the seven most relevant ones for each rating variable (in order not to surpass a threshold of one predictor per 20 observations, given the total of 140 content pages). Because k-fold cross-validated ANNs tend to vary a lot in their results per run, we let each of these analyses run a hundred times with each iteration having a different random seed and averaged the resulting performance (both training and validation) as well as main and total effects for each predictor. The mean total effects of each predictor across these 100 runs were used to select the seven most relevant predictors for each dimension-specific model. We ran another set of ANNs with the exact same approach as for the full models again, but this time for reduced models with only seven predictors each. We did this to make sure that the predictive accuracies of the reduced models were still high enough, and to better understand the role of each of the seven remaining predictors for each rating dimension.

Based on these reduced models, we created bagged predictions for each rating dimension (Breiman, 1996; Hastie et al., 2009) out of 100 bootstrapped samples for a single ANN run with the set of predictors identified for each reduced model. We used these to directly compare predicted (text features) and observed (subjective ratings) values in a linear regression.

Finally, we also looked into the usage of smoothing to account for effects of the immediate narrative context (*cf.* Elkins and Chun, 2019; Jacobs and Kinder, 2019). That is, we ran another set of linear regressions, this time between the moving average of each bagged predictions set and the moving average window of the corresponding rating variable on a supra-page level. For all moving average curves, we applied a window of 5 text units (pages).

To ensure validity and avoid the effects of potential auto-correlation introduced through this smoothing procedure, we used control analyses with permutation, that is, randomly scrambled item orders (*cf.* Crevecoeur et al., 2010; Ali et al., 2022). More precisely, we constructed a null model that the statistical comparisons of these moving average curves could be tested against by averaging the performance of a hundred comparisons between these variables with iteratively randomized page orders for the predictor. In other words, using randomized page orders, we tested if any performance improvements in this step were really due to the smoothing across this specific narrative, and not due to the act of smoothing itself.

As additional sanity check, we also ran additional simple (bivariate) linear regressions for each predictor in each reduced model. These are discussed in detail below, together with the reduced models themselves.

#### Comprehensibility ratings

**Figure 4:**
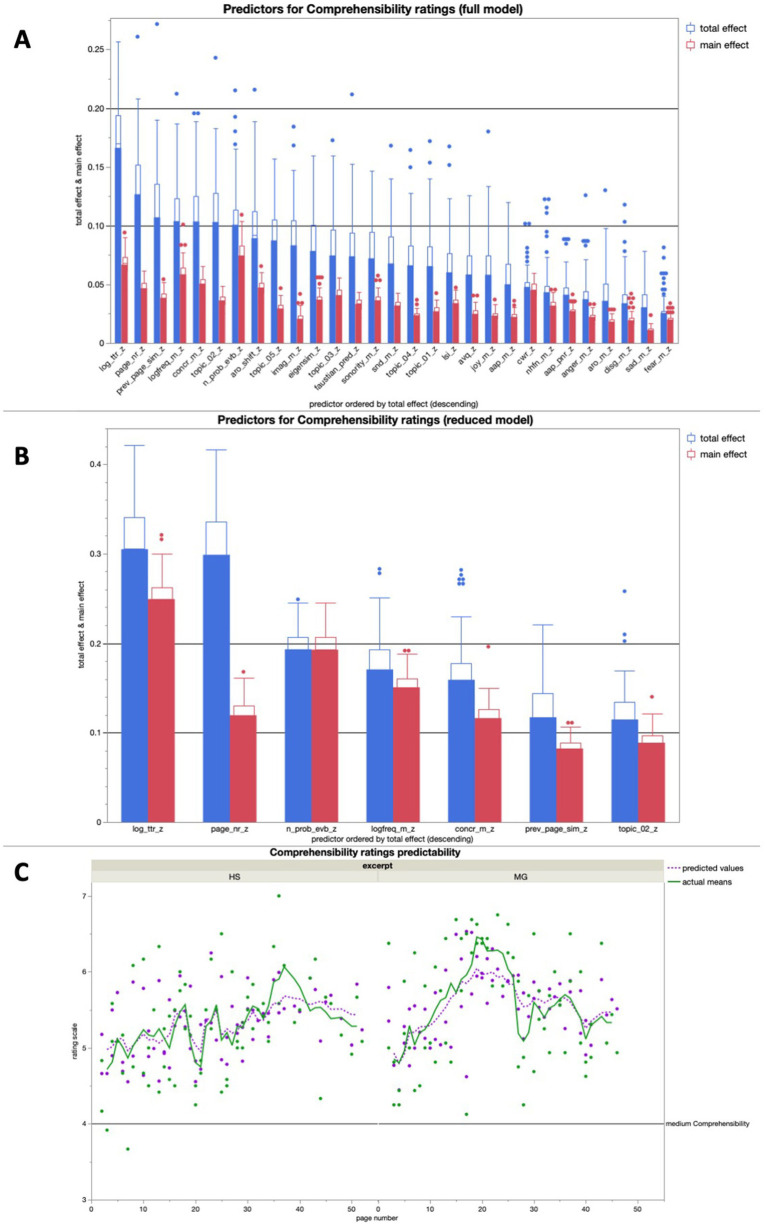
**(A)** Average predictive accuracy of all full model predictors (text features) for the comprehensibility ratings across 100 runs; the seven predictors with the highest average total effect were used for the reduced model for comprehensibility rating predictions; **(B)** Average predictive accuracy of all full model predictors (text properties) for the Comprehensibility ratings across 100 runs; **(C)** Bagged predictions of the reduced model versus smoothed actual values of the comprehensibility ratings; the lines represent supra-page level smoothing that was done with a centered moving average window of 5 for both curves (all analyses were run across both excerpts together).

The full model for comprehensibility averaged high predictive accuracies for both training (*R^2^* = 0.91) and validation (*R^2^* = 0.65) over 100 runs, the most important predictor being *TTR*, the *logarithmic type-token ratio* (Figure 4A). The reduced model, consisting of the seven most important predictors of the full model, averaged a predictive accuracy of *R^2^* > 0.5 for both training (*R^2^* = 0.64) and validation (*R^2^* = 0.57) over 100 runs, the most important predictor again being TTR (Figure 4B).

**Figure 5:**
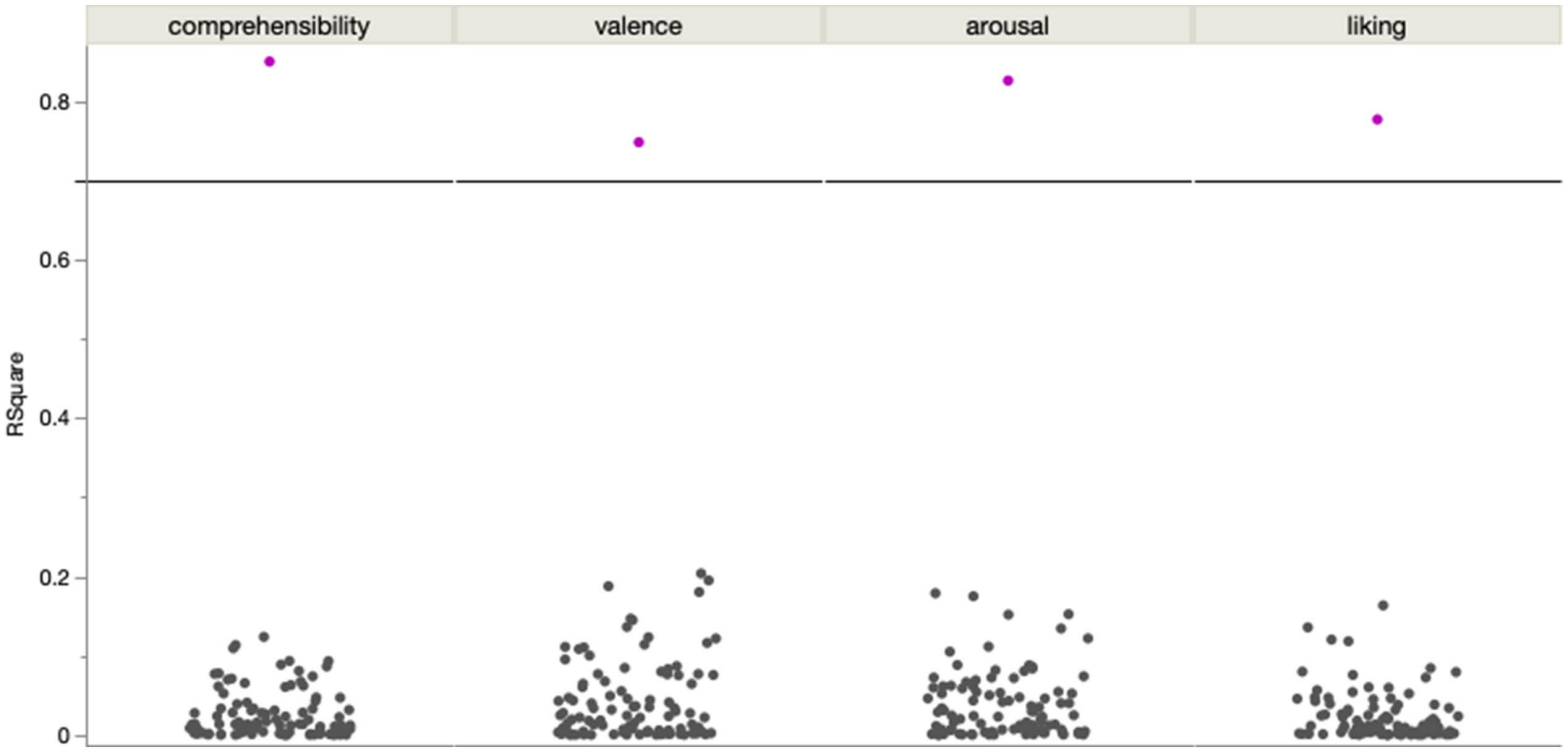
In comparison with 100 permutations of smoothing with different page orders (gray), the smoothing along the original page orders (pink) performed a lot better in its predictive accuracy for the smoothed ratings in all four dimensions.

In a linear regression, the bagged predictions of the reduced model for the comprehensibility ratings showed a high predictive accuracy in a significant positive linear fit [*beta* = 1.327; *t* (1,139) = 19.19; *p* < 0.0001; *R^2^* = 0.73]. After using a moving window average of 5 to smooth both variables on the supra-page level (Figure 4C), the bagged prediction curve showed an even more accurate significant positive linear fit with the Comprehensibility rating curve [*beta* = 1.266; *t* (1,134) = 27.43; *p* < 0.0001; *R^2^* = 0.85], and performed better than all of the control analyses with scrambled page order (Figure 5).

In the additional bivariate linear regressions for each of the predictors in the reduced model for comprehensibility ratings, there were multiple significant relationships. Specifically, the mean logarithmic word frequency, the logarithmic type-token ratio, the mean word concreteness, and the page number each had a positive significant relationship with the comprehensibility ratings, whereas the number of probable event boundaries had a significant negative relationship with the comprehensibility ratings. For an overview of all linear regressions we ran, consult Table 3.

**Table 3:** Simple linear regressions for the individual predictors in all reduced models.

Text feature	Comprehensibility		Valence		Arousal		Liking	
	Beta	*t* ratio	Beta	*t* ratio	Beta	*t* value	Beta	*t* value
logfreq_m	+0.550	*t* = 2.76**						
nhfn_m			−0.094	*t* = −2.16*				
snd_m					+0.042	*t* = 0.02		
log_ttr	+0.595	*t* = 4.89***	−0.068	*t* = −0.44				
sonority_m			+1.829	*t* = 2.88**			−0.046	*t* = −0.11
concr_m	+0.113	*t* = 2.14*						
aap_m			+1.022	*t* = 4.77***				
joy_m			+0.484	*t* = 2.58*				
aro_m					+0.219	*t* = 3.59***		
aro_shift			+0.018	*t* = 0.45			−0.040	*t* = −1.54
n_prob_evb	−0.115	*t* = −3.01**	+0.096	*t* = 2.11*	+0.043	*t* = 0.23	+0.007	*t* = 0.23
topic_02	+1.549	*t* = 1.79			+0.972	*t* = 1.66	+0.777	*t* = 1.18
topic_04					−0.306	*t* = −0.57		
topic_05							−1.313	*t* = −2.41*
prev_page_sim	−0.722	*t* = −1.55			+0.362	*t* = 1.15	−0.0114	*t* = −0.32
page_nr	+0.009	*t* = 2.08*			+0.006	*t* = 1.80	−0.003	*t* = −0.88

Degrees of freedom for all t values: 1, 138; significance markers: *p < 0.05; **p < 0.01; ***p < 0.001; more details about all text features in Appendix 1.

#### Valence ratings

**Figure 6:**
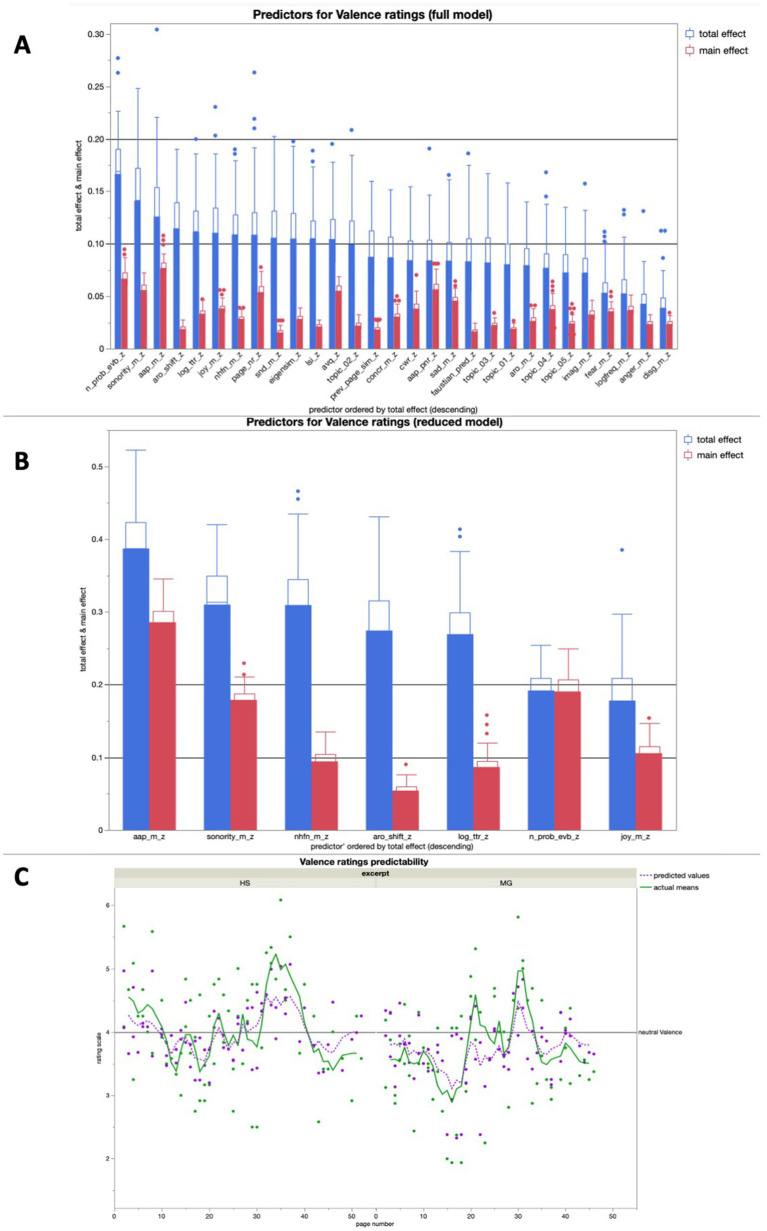
**(A)** Average predictive accuracy of all full model predictors (text properties) for the Valence ratings across 100 runs; the seven predictors with the highest average total effect were used for the reduced model for Valence rating predictions; **(B)** Average predictive accuracy of the seven reduced model predictors (text features) for the valence ratings across 100 runs; **(C)** Bagged predictions of the reduced model versus smoothed actual values of the valence ratings; the lines represent supra-page level smoothing that was done with a centered moving average window of 5 for both curves (all analyses were run across both excerpts together).

The full model for valence averaged high predictive accuracies for both training (*R^2^* = 0.92) and validation (*R^2^* = 0.75). As expected, emotion-related text properties like AAP, *arousal shift*, and *joy potential* all were among the seven most important text features for valence rating prediction in the full model, but the most important one was the *number of probable narrative event boundaries* (Figure 6A). The reduced model for valence averaged a predictive accuracy of *R^2^* > 0.5 for both training (*R^2^* = 0.62) and validation (*R^2^* = 0.59), with AAP being the most important predictor (Figure 6B).

In a linear regression, the bagged predictions of the reduced model for the valence ratings showed a high predictive accuracy in a significant positive linear fit [*beta* = 1.409; *t* (1,139) = 17.71; *p* < 0.0001; *R^2^* = 0.70]. After using a moving window average of 5 to smooth both variables on the supra-page level (Figure 6C), the bagged prediction curve of the reduced model showed an even more accurate significant positive linear fit with the valence rating curve [*beta* = 1.421; *t* (1,134) = 19.86; *p* < 0.0001; *R^2^* = 0.75], and performed better than all of the control analyses with scrambled page order (Figure 5).

In the additional bivariate linear regressions for each of the predictors in the reduced model for valence ratings, there were multiple significant relationships. Specifically, the mean word sonority, the mean word AAP, the mean joy potential, and the number of probable event boundaries each had a positive significant relationship with the valence ratings, whereas the number of higher-frequent orthographic neighbors had a significant negative relationship with the valence ratings (Table 2).

#### Arousal ratings

**Figure 7:**
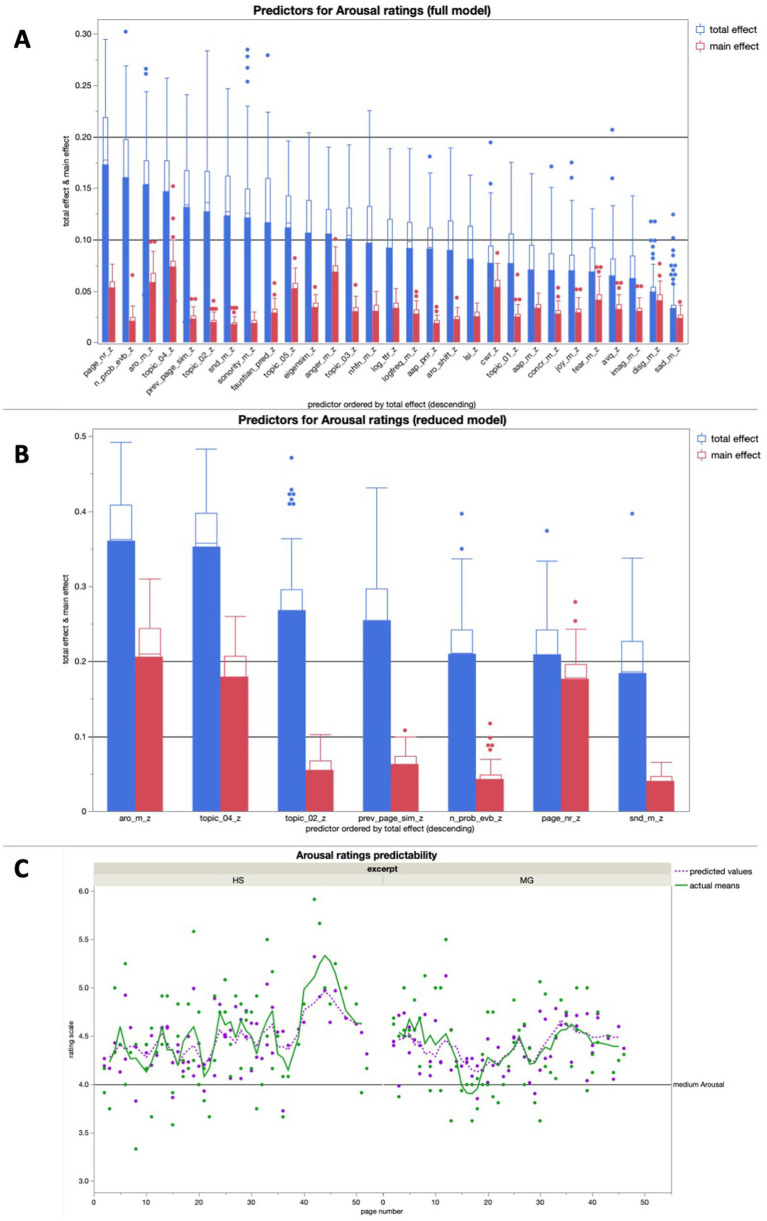
**(A)** Average predictive accuracy of all full model predictors (text features) for the arousal ratings across 100 runs; the seven predictors with the highest average total effect were used for the reduced model for arousal rating predictions; **(B)** Average predictive accuracy of the seven reduced model predictors (text properties) for the Arousal ratings across 100 runs; **(C)** Bagged predictions of the reduced model versus smoothed actual values of the arousal ratings; the lines represent supra-page level smoothing that was done with a centered moving average window of 5 for both curves (all analyses were run across both excerpts together).

The full model for arousal showed high predictive accuracies for both training (*R^2^* = 0.83) and validation (*R^2^* = 0.75) over 100 runs (Figure 7A). The reduced model, consisting of the seven most important predictors of the full model, averaged a predictive accuracy of *R^2^* > 0.5 for both training (*R^2^* = 0.59) and validation (*R^2^* = 0.56) over 100 runs (Figure 7B).

Mean content word arousal potential played a central role in the reduced model for arousal rating predictions, with a total effect *R^2^* > 0.3. The other features in the same model with a predictive accuracy above that threshold is related to a specific story topic, i.e., an immersion-related feature.

In a linear regression, the bagged predictions of the reduced model for the arousal ratings showed a high predictive accuracy in a significant positive linear fit [*beta* = 1.427; *t* (1,139) = 18.68; *p* < 0.0001; *R^2^* = 0.72]. After using a moving window average of 5 to smooth both variables on the supra-page level (Figure 7C), the bagged prediction curve showed an even more accurate significant positive linear fit with the arousal rating curve [*beta* = 1.437; *t* (1,134) = 25.10; *p* < 0.0001; *R^2^* = 0.83], and performed better than all of the control analyses with scrambled page order (Figure 5).

In the additional bivariate linear regressions for each of the predictors in the reduced model for arousal ratings, there was only one significant relationship: The mean arousal potential had a significant positive relationship with the subjective arousal ratings (Table 2).

#### Liking ratings

**Figure 8:**
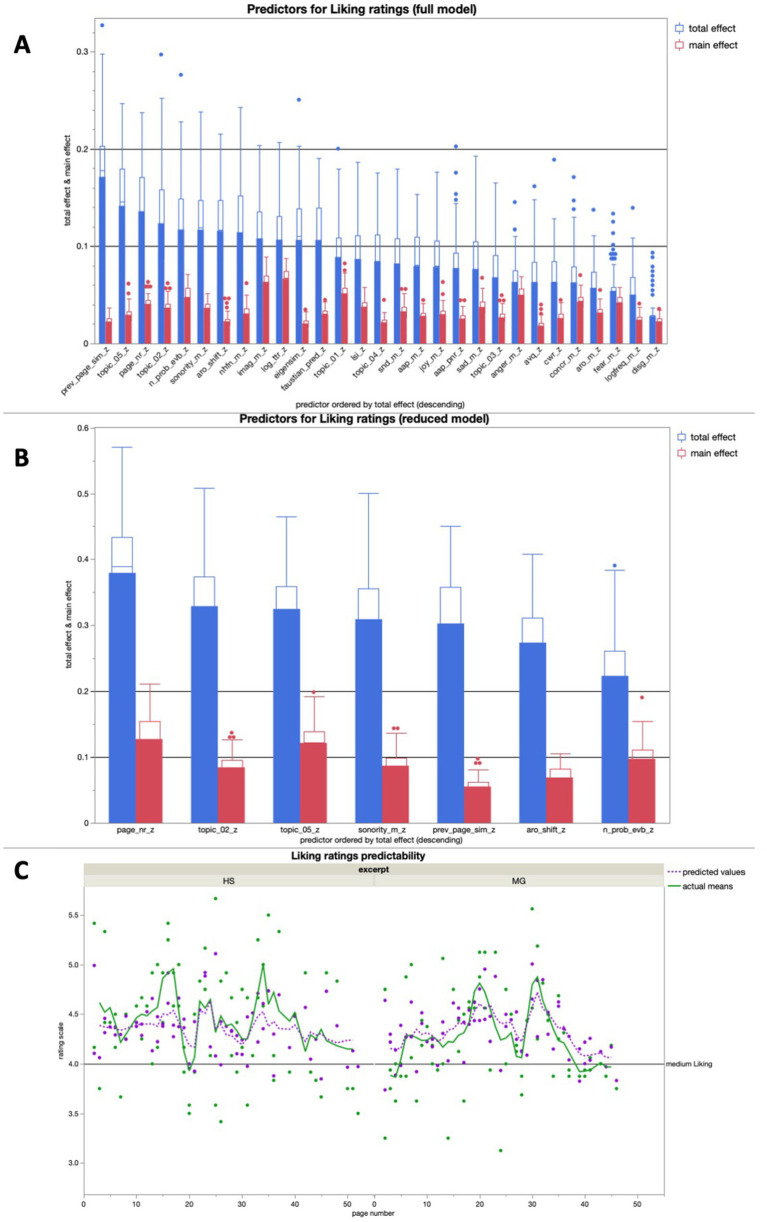
**(A)** Average predictive accuracy of all full model predictors (text features) for the liking ratings across 100 runs; the seven predictors with the highest average total effect were used for the reduced model for liking rating predictions; **(B)** Average predictive accuracy of the 7 reduced model predictors (text properties) for the liking ratings across 100 runs; **(C)** Bagged predictions of the reduced model versus smoothed actual values of the liking ratings; the lines represent supra-page level smoothing that was done with a centered moving average window of 5 for both curves (all analyses were run across both excerpts together).

The full model for liking averaged high predictive accuracies for both training (*R^2^* = 0.82) and validation (*R^2^* = 0.67). The reduced model for liking yielded a predictive accuracy of *R^2^* > 0.5 for both training (*R^2^* = 0.62) and validation (*R^2^* = 0.59). Interestingly, AAP was not among the seven most important features for liking rating prediction in the full model (see Figure 8A).

The on average most important predictors for the Liking ratings in the reduced model were the page number within the excerpt and the respective contribution to two different narrative topics (all three with *R^2^* > 0.3). That being said, all seven predictors in the model had effects of with a stength of *R^2^* > 0.2, making this reduced model more balanced in its predictor importance distribution than the other three reduced models we discussed (see Figure 8B).

In a linear regression, the bagged predictions of the reduced model for the liking ratings showed a high predictive accuracy in a significant positive linear fit [*beta* = 1.630; *t* (1,139) = 18.22; *p* < 0.0001; *R^2^* = 0.71]. After using a moving window average of 5 to smooth both variables on the supra-page level (the lines in Figure 8C), the bagged prediction curve showed an even more accurate significant positive linear fit with the liking rating curve [*beta* = 1.740; *t* (1,134) = 21.51; *p* < 0.0001; *R^2^* = 0.78], and performed better than all of the control analyses with scrambled page order (Figure 5).

In the additional bivariate linear regressions for each of the predictors in the reduced model for liking ratings, there was only one significant relationship: The contribution of a page to topic 5 had a significant positive relationship with the liking ratings (Table 2).

## Discussion

With this study, we investigated the relationship between different subjective rating dimensions of literary reception, as well as the relationship between text features and these rating dimensions over the course of a long work of narrative poetry. More specifically, we asked how the rating dimensions valence, arousal, comprehensibility, and liking relate to each other and if the natural variation of features throughout a long narrative poetry text is useful to predict these dimensions over the course of the narrative. To account for the natural variation between the different features, we ran our analyses on a level of relatively small text units, namely pages of 4 to 12 verse lines. For each text unit, we used the resulting text features to predict the self-reported receptive response the text elicited in readers, in the form of their subjective ratings. Our procedure led to findings in two broader categories, which each need their own discussion in more detail: Findings about the relationships between the four rating dimensions, and findings about the specific text features predicting each rating dimension.

### Rating patterns

The rating patterns alone contain plenty of valuable information. Arousal ratings were independent of the three other dimensions. As shown in Table 3, we observed only two significant relationships between our four rating variables. First, a significant positive correlation between comprehensibility and liking, and second, also a positive correlation between valence and liking. In short, the more comprehensible and positive a page was found to be, the higher its likeability. However, valence and comprehensibility ratings were not related to each other. Thus, they did not appear to interact with each other in predicting liking.

As fuel for the ongoing theoretical discourse about the relationship between comprehensibility and liking, these findings are especially interesting in the context of previous findings regarding the IDEST prose database (Kaakinen et al., 2022). For these short emotional stories, an inversed U-shape best described the relationship between these two constructs (Jacobs, 2023). This suggested that readers enjoy texts which are neither too difficult nor too easy to understand yielding a somewhat optimal level of activation or arousal (Berlyne, 1970; Errington et al., 2022). There is the possibility that an 19th century work of poetic literature is by its nature hard enough to read that the rightmost end of the inverse U-shape is not visible, that is, the (too) high comprehensibility leading to low-likeability part of the U-shape does not apply when a text is just not that comprehensible. However, the counter-argument would be that the average comprehensibility of the text excerpts was overall rated rather highly. In addition to that, the U-curve has been linked to individual differences before (Güçlütürk et al., 2016), which of course is an aspect of literary processing that we consciously put aside with our average-based approach to this study.

Alternatively, we May be observing a peculiarity of narrative poetry here, where liking and comprehensibility interact differently than in regular prose. The Pleasure-Interest model of aesthetic liking (Graf and Landwehr, 2015) argues that several processing filters apply during literary reading, depending on a balance between the need for cognitive enrichment (which influences liking) and the cognitive effort needed to achieve reading fluency (which depends on comprehensibility). It could be that the combined complexity of processing both poetry and a long narrative at the same time adds an additional layer of effort on top of the regularly varying comprehensibility in a text. In that case, the balance between effort and enrichment would be tilted toward effort, and any text passages that are higher in comprehensibility would linearly lead to higher liking.

The concept of a reliance of high-dimensional enjoyment on basic emotions would mean that the extreme instances of valence (i.e., both negative and positive) lead to higher liking ratings than flat (close-to-neutral) valence (Jacobs et al., 2016; Kraxenberger and Menninghaus, 2017). However, the relationship between valence and liking ratings was also linear and positive. The correlation between positive valence and high liking was thus expected, but it is remarkable that there was none between high liking and negative valence. A simple possible explanation could be the stimulus material itself: All of it being part of one single piece of work might just not include the full spectrum of valence that makes its varied relationship with liking visible in multiple-piece poetry studies.

Overall, the four chosen rating dimensions thus seem to cover four different aspects of the reader response. A positive Valence potential is better-liked by the reader, whereas high and low Arousal are emotional amplifications on an axis that is independent of Valence and Liking (*cf.* Russell and Barrett, 1999). The latter can also be regarded as an indication for an extended role of the aesthetic processing trajectory for the subjective Liking of narrative poetry. We could then state that literary appreciation based on Valence can be linked to the processing of poetry (*cf.* Jacobs and Kinder, 2019), whereas Arousal-based appreciation is typically linked to the processing of narratives (*cf.* Kaakinen and Simola, 2020).

### Text features

All four rating dimensions were predicted very accurately by the full set of 29 features, all of them having training and validation *R^2^*s > = 0.65. Just like the rating patterns showed differences between the rating dimensions, there were also differences in which text features were more important for predicting each rating dimension. Reducing the full set to only seven features per rating dimension gave us a lot of additional information about which aspects of the text related to which rating dimension. Fewer predictors obviously lead to a performance drop, but each reduced set of features still performed fairly accurately, all of them having training and validation *R^2^*s > 0.5.

The additional simple bivariate linear regressions gave more insights into the directions of effect for some of the individual text features as predictors for each of the four rating dimensions. However, some text features did not have an observable significant relationship with a rating dimension individually, even when they did contribute to the predictive model in the ANN analyses (Table 2; Figures 4B, 6B, 7B, 8B). This showcases one of the main advantages of ANN analyses for literary text data: They allow us to account for non-linear dependencies and non-linear interactions within the data that we get from our multi-dimensional text profiling tools (Figure 1). These same text profiling tools and ANN analyses also showed us that there is a specific set of text features tied to each rating dimension (our reduced models). While these sets partially overlap, there was only one text feature that appeared as a predictor in all four reduced models: The number of probable narrative event boundaries on a page (Table 2). This text feature specifically, and the narrative segmentation aspect it represents in literary processing, certainly proved worthy of more attention in future research.

In sum, the average reader response on the subjective-experiential level was largely predictable with purely text-based predictors. In this study, the four rating dimensions comprehensibility, valence, arousal, and liking could well be predicted with only seven features each. We thus successfully replicated findings of previous studies using text analysis tools to predict reader responses (Crossley et al., 2017; Ullrich et al., 2017; Jacobs and Kinder, 2019; Xue et al., 2020; Lüdtke and Hugentobler, 2022; Xue et al., 2023; Mak et al., 2023; Lei et al., 2023) and extended them to ratings of narrative poetry, as well as to the broadest range of reader response dimensions so far. In addition to that, we used theoretically motivated text features rather than unsupervised deep-learning for our predictive models. As a result, we can further dissect the predictive models for each rating dimension to better understand which textual cues our readers used to evaluate their reading experience and accordingly rate the four dimensions. Such an approach can even explain which particular feature set was likely most influential for a given individual reader (Jacobs, 2023).

#### Text features predicting comprehensibility ratings

Regarding comprehensibility ratings, we expected surface text features to be the most important ones, that is, aspects of the text that are more related to its immediate readability than its semantic content. And indeed, the single most important predictor of comprehensibility ratings in both the full and the reduced models (Figures 4A,B) was the TTR. This feature indexes lexical diversity within a text unit, i.e., the number of repetitions of words that occur. This was thus an expected result and supports basic concepts on text readability (Graesser et al., 2004; Kettunen, 2014).

Interestingly, out of the six remaining predictors in the reduced model, only one other can be strictly defined as a surface text feature, namely log word frequency, which is also known to contribute to low-level (=surface) readability (Crossley et al., 2011; Smith and Levy, 2013; Xue et al., 2020). Two predictors in the reduced model were clearly related to the narrative, namely the number of probable narrative event boundaries on a page, as well as a page’s contribution to the semantic topic 2. Two other predictors were calculated using semantic networks, but arguably measure text readability, albeit on a higher level, namely the average content word concreteness (Sadoski et al., 2000) and the semantic similarity to the previous page, possibly operating as a measure of cohesion here (*cf.* Graesser et al., 2004).

Finally, the page number within the excerpt came in as second-most important predictor in the reduced model of comprehensibility ratings, with a total effect that was merely lower than the one of TTR, but with a much lower main effect. The latter suggests that much of the page number’s total effect came from interactions with the other predictors. The page number restarted counting for each excerpt, making it unlikely that this is a pure effect of readers getting tired over the course of the experiment. In that light, it seems possible that the total effect of the page number (also) reflects a narrative dimension of understanding, in the sense that the position of a page within an excerpt always also reflects the position of a page’s content within the narrative structure.

Either way, comprehensibility ratings seem to be linked to both surface text readability and a higher-level narrative understanding. The additional linear regressions underline this, showing that surface text features lead to higher comprehensibility ratings, but a large number of probable event boundaries lead to lower comprehensibility ratings. Surface text readability has been shown to play an important role for pure poetry processing (Xue et al., 2020; Xue et al., 2023), so it is reassuring that related text features also played a role for the comprehensibility of our poetic stimulus material, suggesting an involved aesthetic trajectory. At the same time, the importance of text features related to higher-level narrative understanding indicates that the immersive trajectory was also involved with the comprehensibility ratings. This could mean that there are two separate types of comprehensibility affecting ratings, one of which is related to lower-level surface understanding and aesthetic processing, and the other one to higher-level narrative understanding and immersive processing. Such a hypothesis is best tested with neuroimaging methodology, since the immersive and aesthetic trajectories manifest themselves most clearly on the neural level (Hartung et al., 2021; Jacobs, 2023).

#### Text features predicting valence ratings

We expected AAP to be the single most important predictor of valence ratings, given that it reflects both the potential for emotional positivity and the usage of stylistic beauty in a text (Jacobs, 2017, 2023; Jacobs and Kinder, 2019). We had associated both of these aspects to the aesthetic processing trajectory and found that AAP was indeed (I) the most important predictor in the reduced model of valence ratings (Figure 6B), (II) among the best few in the full model in terms of the total effect, and (III) had the single highest main effect (Figure 6A). The next best predictor in the reduced model was the average content word *sonority*, a feature reflecting both euphony and pronounceability (Vennemann, 1988, as cited in Jacobs, 2017; Xue et al., 2020), which thus highlights both text readability and the poetic element of narrative poetry.

Another predictor in the reduced model for the valence ratings that can be related to the aesthetic processing trajectory is *SentiArt*’s average content word *joy* potential, the most positive of the five basic emotions included in the full model and a feature that has been linked to elementary affective decisions and liking before (Dodds et al., 2011; Jacobs et al., 2016). Joy potential, sonority, and AAP also showed significant positive relationships with the valence ratings on their own in our additional linear regression analyses. All of these findings fit well with the concept of beauty appreciation, positive valence, and an aesthetic processing trajectory being closely linked with one another. The *number of higher frequency neighbors* (NHFN; Grainger and Jacobs, 1996) and TTR are both surface features, and their importance in predicting the valence ratings reinforces the idea that comprehensibility in poetry processing heavily depends on surface features (Xue et al., 2020, 2023). Given the independent linear alignment of both valence and comprehensibility ratings with liking ratings, it seems plausible that low-level surface understanding and the appreciation of positive valence both contribute to the likeability of a poetic text. Indeed, NHFN also showed an accordingly negative significant relationship with the valence ratings on its own in our additional linear regression analysis.

The other two predictors in the reduced model for valence ratings are the *arousal shift*, i.e., the difference between consecutive pages in terms of the average content word arousal, and the *number of probable narrative event boundaries*. Given their direct links to emotional arcs (*cf.* Reagan et al., 2016) and narrative structure (Radvansky and Zacks, 2017; Geerligs et al., 2021), respectively, we would view both of them as related mainly to the immersive processing trajectory. The positive significant relationship of the number of probable event boundaries on its own with the valence ratings that we found in our additional linear regression analysis implies that more narrative density leads to higher valence ratings. This also fits well with the dysfluency prediction for the aesthetic trajectory of the NCPM: More narrative density leads to more information to process and therefore less reading fluency, which co-occurs with higher valence ratings.

The reader’s emotional evaluation of narrative poetry in terms of valence thus seems to depend on both the textual esthetics of the poetry, and the immersive content of the narrative. Of course, it could also just be that the immersive trajectory is stronger than the aesthetic trajectory in narrative poetry reading, thereby also “leaking” into this rating dimension. To further investigate this, we are currently conducting research with methodology that allows us to explore the neurocognitive underpinnings of these processing trajectories.

#### Features predicting arousal ratings

Two aspects are remarkable about the reduced prediction model for arousal ratings (Figure 7B). First, GLEAN’s arousal potential is the most important predictor, neatly validating this specific text analysis tool. The arousal potential also showed the only significant relationship with the valence ratings on its own in our additional linear regression analysis – not so surprisingly, a positive one. The model also stands out because five of the remaining six features can be directly associated with the narrative; the only exception being *semantic neighborhood density* (SND). This fits perfectly with the notion that arousal ratings serve as a window to the immersive processing trajectory, through the detour of suspense correlations (*cf.* Wallentin et al., 2011; Kaakinen and Simola, 2020; Jacobs et al., 2016; Jacobs and Lüdtke, 2017). To be clear, as argued above, specifically the features page number and semantic similarity to the previous page probably do not only relate to the narrative, but also to certain aspects of comprehensibility. However, these are aspects of comprehensibility that do not relate to the text surface, but to higher-level understanding. Given its decidedly semantic aspects, this is also true for SND. Arousal ratings are thus clearly dependent on higher-level processing and likely to be related to the immersive processing trajectory.

#### Features predicting liking ratings

In the prediction of liking ratings, the seven predictors of the reduced model were somewhat close to each other in their average importance, creating a rather flat comparison of total effects, and generally rather low main effects (Figure 8B). This suggest that many aspects of text processing have to be accounted for when predicting liking ratings (*cf.* Jacobs et al., 2016; Jacobs, 2023), including individual differences (Jacobs, 2023; Lei et al., 2023). Using the same logic, we applied in the interpretation of the other reduced models, we can observe four features that clearly relate to the immersive processing trajectory, namely arousal shift, the number of probable narrative event boundaries, and the respective contributions to two different semantic topics. Interestingly, one of these two topics also showed the only significant relationship with the liking ratings on its own in our additional linear regression analyses – and it was a negative one: Our subject seemed to have particularly liked this on specific topic. Next to that, we find two features that have proven to be rather hard to classify between comprehensibility and immersion so far, namely page number in the excerpt, and semantic similarity to the previous page. It can thus be stated without a doubt that text features related to higher-level narrative understanding played an important role for our liking ratings.

According to previous research AAP should also be an important predictor for narrative liking (Jacobs and Kinder, 2019). However, this was not the case in our dataset. At the same time, the features we did observe to play a role for the liking ratings were still related to the narrative. The liking ratings were also linearly aligned with the comprehensibility ratings, and both dimensions share four out of seven predictors in their reduced models. Then again, the liking ratings were also linearly aligned with the valence ratings, but not with the arousal ratings. With all of that in mind, the complexity of liking ratings for narrative poetry cannot be overstated. If there are separate processing trajectories for immersive and aesthetic aspects of literary reception, they at the very least interact in the emergence of liking, if nowhere else.

Just like for arousal ratings, surface readability also seems to play less of a role for the liking ratings than higher-level comprehension does. In that regard, liking ratings for narrative poetry mostly seem to depend on higher-level processing. That being said, the sonority score feature can be linked to both pronounceability (and therefore readability) and aesthetic processing. Higher-level comprehension, the immersive processing trajectory, and sonority – possibly related to the poetic aspects of the text – are thus the central elements that predict liking ratings for narrative poetry, but not the basic emotions and stylistics that we expected to be important based on previous studies (Simonton, 1990; Delmonte, 2016; Jacobs et al., 2016). Again, liking ratings seem rather complex in their nature, which makes sense, given the complexity of our stimulus material, and the fact that we ignored reader-specific data in this study.

#### Outlook

The supra-page smoothing did clearly improve the predictive accuracy for all four reduced models in comparison to the page-level analyses, even when compared to the already performance-improved bagged predictions (*cf.* Reagan et al., 2016; Jacobs and Kinder, 2019). The permutation analyses showed that these are not effects of the smoothing itself, but effects directly related to this specific order of pages that constitute the narrative structure. Interestingly, this implies that there are effects of the larger narrative on the rating behavior that we could model with our page-based text analyses. That being said, some of our page-based features related to the larger narrative to begin with. Thus, our findings also serve as a reminder to consider various levels of text features when predicting reader responses. This of course is especially relevant for future research on other methodological levels: For instance, eye-tracking has a higher temporal resolution for reading research and provides reader response data even on the sub-lexical level. However, researchers limiting analyses of eye-tracking studies on literary reading to such a detailed level might for example miss possible effects of higher-level comprehensibility on reading speed.

Of course, there were also limitations to our approach. This study’s stimulus material is all taken from the same literary work. This was prudent in the sense that it allowed us to do comparisons between pages from one coherent text body, which freed us from having to account for idiosyncratic differences between different texts. However, it also limits the generalizability of our results to other the processing of other literary texts now. In the same vein, using stimulus material of the genre of narrative poetry allowed us to compare processing trajectories that would not interact so clearly in other text types, but it again limits the generalizability of our results for other genres. After all, different text types are also received differently by the reader (Quezada Gaponov et al., 2024).

We would like to claim that we investigated the processing of a naturalistic text in an experimental setting, but in reality, we had to walk a fine line in deciding where to compromise on which of those two aspects. Subjects were paid to read a certain text at a certain time, and did so in an experimental setting, constantly interrupting their reading flow to rate each page. All of this could be argued to be detrimental to a true naturalistic reading experience, but was necessary for experimental control. The subjects also read on a digital screen rather than on paper, although this hardly seems to be a limitation in terms of the reading experience (Sorrentino, 2021).

Finally, it is worth mentioning that for this study, we focused on the average reader response to literary texts rather than individual differences between readers in their responses. In our view, this was a necessary first step in this specific niche of empirical literary studies for now. We considered it worthwhile to first identify common denominators in the cognitive processes underlying literary reading before looking into individual nuances influencing these processes. The focus on the average reader allowed us to use sophisticated multivariate analyses rather than simpler mixed models, thereby creating a stronger theoretical base for future research, hopefully including many individual differences studies.

Now that we have a better insight into which text features drive which aspects of direct-subjective reader responses, it is easier to conduct more much-needed research on the indirect-objective level to find out more about the interaction between different processing trajectories, specifically with other behavioral and neuroimaging methods. Especially neuroimaging has proven to function as a detailed window into naturalistic narrative reception processes (Altmann et al., 2012, 2014; Hsu et al., 2015; Lehne et al., 2015; Geerligs et al., 2021; Le et al., 2022; Oetringer et al., 2024).

## Conclusion

To summarize, it seems clear that the average subjective reader response, as sampled in the rating dimensions comprehensibility, valence, arousal, and liking, can successfully be predicted by quantitative text features, even if the text is a multi-layered and complex one like a work of narrative poetry. The multivariate ANN models all achieved high prediction accuracies, revealing the crucial aspects of reader’s text processing that influence their experiential judgments. Valence ratings were affected by features associated with both esthetics and narrative immersion, arousal ratings mostly by those associated with narrative immersion, and comprehensibility ratings by both surface features and high-level narrative aspects of the texts.

The possibly hardest to interpret results were obtained for liking ratings, which were predicted by a broad variety of as features, including those associated with narrative immersion and high-level comprehensibility. The more complex a text is, the more complex it seems to be to accurately predict how much subjects will like it (*cf.* Jacobs, 2015, 2023; Jacobs and Willems, 2018; Willems and Jacobs, 2016). That notwithstanding, we conclude that liking ratings for narrative poetry are mostly a result of the reader’s evaluation of a text’s comprehensibility, structural cohesion, and narrative content. In comparison, the role of aesthetic and word-level emotion potentials in this text evaluation is less clear. At present, we interpret this as readers treating narrative poetry more like a story than like a poem – although both aspects are present in the reader response.

In any case, it is clear that a sort of higher-level processing affected all rating dimensions through narrative structures, likely similar to the immersive processing trajectory of the NCPM. The NCPM’s aesthetic processing trajectory was less dominant in our subjective reader responses to narrative poetry, but it seemed to have played a role, given the observed importance of the AAP feature for predicting valence ratings. Comprehensibility does not seem to be strictly linked to the immersive trajectory, at least for the average reader. Rather, there seems to be a distinction between higher- and surface-level processing as well, possibly also related to both trajectories of the NCPM. If so, liking with its high linear correlation with both comprehensibility and emotional text aspects seems to be a potential window into the interaction between both trajectories that in most realistic reading scenarios do not exclude each other (Jacobs, 2021, 2023). The Liking ratings thereby also have proven to be heavily dependent on comprehensibility, but not exclusively, which fits nicely with the theoretical implications of the pleasure-interaction model of aesthetic liking (Graf and Landwehr, 2015). Future research using neuroimaging will help to account better for these nuances.

## Data Availability

The raw data supporting the conclusion of this article are available from the corresponding author on request.

## Footnotes

^1^ https://www.soscisurvey.de/
