# Supplementary material for: Predicting subjective ratings of affect and comprehensibility with text features: a reader response study of narrative poetry
Source: Front Psychol. 2024 Oct 8;15:1431764. doi: 10.3389/fpsyg.2024.1431764 (PMC11494826; doi:10.3389/fpsyg.2024.1431764)
Supplement: Supplementary file 1 [file Table_1.pdf]

Appendix 1: Stimulus material

excerpt	stanza_nr	stanza_content	scene_nr	scene
hs1	1	*Im Himmel.*<br>*Erzengel tritt vor.*<br><br>"Die Sonne tönt, nach alter Weise,<br>In Brudersphären Wettgesang,<br>Und ihre vorgeschriebne Reise<br>Vollendet sie mit Donnergang.<br>Ihr Anblick gibt den Engeln Stärke,<br>Wenn keiner Sie ergründen mag;<br>die unbegreiflich hohen Werke<br>Sind herrlich wie am ersten Tag."	1	Prolog.3
hs1	2	*Erzengel Gabriel tritt vor.*<br><br>"Und schnell und unbegreiflich schnelle<br>Dreht sich umher der Erde Pracht;<br>Es wechselt Paradieseshelle<br>Mit tiefer, schauervoller Nacht.<br>Es schäumt das Meer in breiten Flüssen<br>Am tiefen Grund der Felsen auf,<br>Und Fels und Meer wird fortgerissen<br>Im ewig schnellem Sphärenlauf."	1	Prolog.3

context marked in yellow

excerpt	stanza_nr	stanza_content	scene_nr	scene
hs1	3	*Der dritte Erzengel, Michael, tritt vor.*<br><br>"Und Stürme brausen um die Wette<br>Vom Meer aufs Land, vom Land aufs Meer,<br>und bilden wütend eine Kette<br>Der tiefsten Wirkung rings umher.<br>Da flammt ein blitzendes Verheeren<br>Dem Pfade vor des Donnerschlags.<br>Doch deine Boten, Herr, verehren<br>Das sanfte Wandeln deines Tags."	1	Prolog.3
hs1	4	*Nun alle drei zusammen.*<br><br>"Der Anblick gibt den Engeln Stärke,<br>Da keiner dich ergründen mag,<br>Und alle deine hohen Werke<br>Sind herrlich wie am ersten Tag."	1	Prolog.3

context marked in yellow

excerpt	stanza_nr	stanza_content	scene_nr	scene
hs1	5	*Es folgt Mephistopheles.*<br><br>"Da du, o Herr, dich einmal wieder nahst<br>Und fragst, wie alles sich bei uns befinde,<br>Und du mich sonst gewöhnlich gerne sahst,<br>So siehst du mich auch unter dem Gesinde.<br>Verzeih, ich kann nicht hohe Worte machen,<br>Und wenn mich auch der ganze Kreis verhöhnt;<br>Mein Pathos brächte dich gewiß zum Lachen,<br>Hättst du dir nicht das Lachen abgewöhnt.<br>Von Sonn' und Welten weiß ich nichts zu sagen,<br>Ich sehe nur, wie sich die Menschen plagen.<br>Der kleine Gott der Welt bleibt stets von gleichem Schlag,<br>Und ist so wunderlich als wie am ersten Tag.	1	Prolog.3
hs1	6	Ein wenig besser würd er leben,<br>Hättst du ihm nicht den Schein des Himmelslichts gegeben;<br>Er nennt's Vernunft und braucht's allein,<br>Nur tierischer als jedes Tier zu sein.<br>Er scheint mir, mit Verlaub von euer Gnaden,<br>Wie eine der langbeinigen Zikaden,<br>Die immer fliegt und fliegend springt<br>Und gleich im Gras ihr altes Liedchen singt;<br>Und läg er nur noch immer in dem Grase!<br>In jeden Quark begräbt er seine Nase."	1	Prolog.3

context marked in yellow

excerpt	stanza_nr	stanza_content	scene_nr	scene
hs1	7	*Der Herr im Himmel ergreift das Wort. Mephistopheles antwortet ihm.*<br>*Es entsteht ein Gespräch zwischen den beiden.*<br><br>"Hast du mir weiter nichts zu sagen?<br>Kommst du nur immer anzuklagen?<br>Ist auf der Erde ewig dir nichts recht?"<br>"Nein Herr! ich find es dort, wie immer, herzlich schlecht.<br>Die Menschen dauern mich in ihren Jammertagen,<br>Ich mag sogar die armen selbst nicht plagen."<br>"Kennst du den Faust?" "Den Doktor?" "Meinen Knecht!"	1	Prolog.3
hs1	8	"Fürwahr! er dient Euch auf besondre Weise.<br>Nicht irdisch ist des Toren Trank noch Speise.<br>Ihn treibt die Gärung in die Ferne,<br>Er ist sich seiner Tollheit halb bewußt;<br>Vom Himmel fordert er die schönsten Sterne<br>Und von der Erde jede höchste Lust,<br>Und alle Näh und alle Ferne<br>Befriedigt nicht die tiefbewegte Brust."	1	Prolog.3
hs1	9	"Wenn er mir auch nur verworren dient,<br>So werd ich ihn bald in die Klarheit führen.<br>Weiß doch der Gärtner, wenn das Bäumchen grünt,<br>Das Blüt und Frucht die künft'gen Jahre zieren."	1	Prolog.3

context marked in yellow

excerpt	stanza_nr	stanza_content	scene_nr	scene
hs1	10	"Was wettet Ihr? den sollt Ihr noch verlieren!<br>Wenn Ihr mir die Erlaubnis gebt,<br>Ihn meine Straße sacht zu führen."<br>"Solang er auf der Erde lebt,<br>So lange sei dir's nicht verboten,<br>Es irrt der Mensch so lang er strebt."	1	Prolog.3
hs1	11	"Da dank ich Euch; denn mit den Toten<br>Hab ich mich niemals gern befangen.<br>Am meisten lieb ich mir die vollen, frischen Wangen.<br>Für einem Leichnam bin ich nicht zu Haus;<br>Mir geht es wie der Katze mit der Maus."	1	Prolog.3
hs1	12	"Nun gut, es sei dir überlassen!<br>Zieh diesen Geist von seinem Urquell ab,<br>Und führ ihn, kannst du ihn erfassen,<br>Auf deinem Wege mit herab,<br>Und steh beschämt, wenn du bekennen mußt:<br>Ein guter Mensch, in seinem dunklen Drange,<br>Ist sich des rechten Weges wohl bewußt."	1	Prolog.3
hs1	13	"Schon gut! nur dauert es nicht lange.<br>Mir ist für meine Wette gar nicht bange.<br>Wenn ich zu meinem Zweck gelange,<br>Erlaubt Ihr mir Triumph aus voller Brust.<br>Staub soll er fressen, und mit Lust,<br>Wie meine Muhme, die berühmte Schlange."	1	Prolog.3

context marked in yellow

excerpt	stanza_nr	stanza_content	scene_nr	scene
hs1	14	"Du darfst auch da nur frei erscheinen;<br>Ich habe deinesgleichen nie gehaßt.<br>Von allen Geistern, die verneinen,<br>ist mir der Schalk am wenigsten zur Last.<br>Des Menschen Tätigkeit kann allzu leicht erschlaffen,<br>er liebt sich bald die unbedingte Ruh;<br>Drum geb ich gern ihm den Gesellen zu,<br>Der reizt und wirkt und muß als Teufel schaffen.	1	Prolog.3
hs1	15	Doch ihr, die echten Göttersöhne,<br>Erfreut euch der lebendig reichen Schöne!<br>Das Werdende, das ewig wirkt und lebt,<br>Umfass euch mit der Liebe holden Schranken,<br>Und was in schwankender Erscheinung schwebt,<br>Befestigt mit dauernden Gedanken!"	1	Prolog.3
hs1	16	*Der Himmel schließt sich, die Erzengel gehen ihrer Wege.*<br>*Mephistopheles ist jetzt alleine.*<br><br>"Von Zeit zu Zeit seh ich den Alten gern,<br>Und hüte mich, mit ihm zu brechen.<br>Es ist gar hübsch von einem großen Herrn,<br>So menschlich mit dem Teufel selbst zu sprechen."	1	Prolog.3

context marked in yellow

excerpt	stanza_nr	stanza_content	scene_nr	scene
hs1	17	*Es ist Nacht.*<br>*In einem hochgewolbtem, engen Zimmer sitzt Faust unruhig an seinem Pult.*<br><br>"Habe nun, ach! Philosophie,<br>Juristerei und Medizin,<br>Und leider auch Theologie<br>Durchaus studiert, mit heißem Bemühn.<br>Da steh ich nun, ich armer Tor!<br>Und bin so klug als wie zuvor;<br>Heiße Magister, heiße Doktor gar<br>Und ziehe schon an die zehen Jahr<br>Herauf, herab und quer und krumm<br>Meine Schüler an der Nase herum-<br>Und sehe, daß wir nichts wissen können!<br>Das will mir schier das Herz verbrennen.	2	Nacht.1
hs1	18	Zwar bin ich gescheiter als all die Laffen,<br>Doktoren, Magister, Schreiber und Pfaffen;<br>Mich plagen keine Skrupel noch Zweifel,<br>Fürchte mich weder vor Hölle noch Teufel-<br>Dafür ist mir auch alle Freud entrissen,<br>Bilde mir nicht ein, was Rechts zu wissen,<br>Bilde mir nicht ein, ich könnte was lehren,<br>Die Menschen zu bessern und zu bekehren.	2	Nacht.1

context marked in yellow

excerpt	stanza_nr	stanza_content	scene_nr	scene
hs1	19	Auch hab ich weder Gut noch Geld,<br>Noch Ehr und Herrlichkeit der Welt;<br>Es möchte kein Hund so länger leben!<br>Drum hab ich mich der Magie ergeben,<br>Ob mir durch Geistes Kraft und Mund<br>Nicht manch Geheimnis würde kund;<br>Daß ich nicht mehr mit saurem Schweiß<br>Zu sagen brauche, was ich nicht weiß;<br>Daß ich erkenne, was die Welt<br>Im Innersten zusammenhält,<br>Schau alle Wirkenskraft und Samen,<br>Und tu nicht mehr in Worten kramen.	2	Nacht.1
hs1	20	O sähst du, voller Mondenschein,<br>Zum letztenmal auf meine Pein,<br>Den ich so manche Mitternacht<br>An diesem Pult herangewacht:<br>Dann über Büchern und Papier,<br>Trübsel'ger Freund, erschienst du mir!<br>Ach! könnt ich doch auf Bergeshöhn<br>In deinem lieben Lichte gehn,<br>Um Bergeshöhle mit Geistern schweben,<br>Auf Wiesen in deinem Dämmer weben,<br>Von allem Wissensqualm entladen,<br>In deinem Tau gesund mich baden!	3	Nacht.2

context marked in yellow

excerpt	stanza_nr	stanza_content	scene_nr	scene
hs1	21	Weh! steck ich in dem Kerker noch?<br>Verfluchtes dumpfes Mauerloch,<br>Wo selbst das liebe Himmelslicht<br>Trüb durch gemalte Scheiben bricht!<br>Beschränkt mit diesem Bücherhauf,<br>den Würme nagen, Staub bedeckt,<br>Den bis ans hohe Gewölb hinauf<br>Ein angeraucht Papier umsteckt;<br>Mit Gläsern, Büchsen rings umstellt,<br>Mit Instrumenten vollgepfropft,<br>Urväter Hausrat drein gestopft-<br>Das ist deine Welt! das heißt eine Welt!	3	Nacht.2
hs1	22	Und fragst du noch, warum dein Herz<br>Sich bang in deinem Busen klemmt?<br>Warum ein unerklärter Schmerz<br>Dir alle Lebensregung hemmt?<br>Statt der lebendigen Natur,<br>Da Gott die Menschen schuf hinein,<br>Umgibt in Rauch und Moder nur<br>Dich Tiergeripp und Totenbein.	3	Nacht.2

context marked in yellow

excerpt	stanza_nr	stanza_content	scene_nr	scene
hs1	23	Flieh! auf! hinaus ins weite Land!<br>Und dies geheimnisvolle Buch,<br>Von Nostradamus' eigner Hand,<br>Ist dir es nicht Geleit genug?<br>Erkennest dann der Sterne Lauf,<br>Und wenn Natur dich Unterweist,<br>Dann geht die Seelenkraft dir auf,<br>Wie spricht ein Geist zum andren Geist.<br>Umsonst, daß trocknes Sinnen hier<br>Die heil'gen Zeichen dir erklärt:<br>Ihr schwebt, ihr Geister, neben mir;<br>Antwortet mir, wenn ihr mich hört!"	3	Nacht.2
hs1	24	*Faust schlägt ein Buch auf und erblickt das Zeichen des Makrokosmos.*<br><br>"Ha! welche Wonne fließt in diesem Blick<br>Auf einmal mir durch alle meine Sinnen!<br>Ich fühle junges, heil'ges Lebensglück<br>Neuglühend mir durch Nerv' und Adern rinnen.<br>War es ein Gott, der diese Zeichen schrieb,<br>Die mir das innre Toben stillen,<br>Das arme Herz mit Freude füllen,<br>Und mit geheimnisvollem Trieb<br>Die Kräfte der Natur rings um mich her enthüllen?	4	Nacht.3

context marked in yellow

excerpt	stanza_nr	stanza_content	scene_nr	scene
hs1	25	Bin ich ein Gott? Mir wird so licht!<br>Ich schau in diesen reinen Zügen<br>Die wirkende Natur vor meiner Seele liegen.<br>Jetzt erst erkenn ich, was der Weise spricht:<br>Die Geisterwelt ist nicht verschlossen;<br>Dein Sinn ist zu, dein Herz ist tot!<br>Auf, bade, Schüler, unverdrossen<br>Die ird'sche Brust im Morgenrot!"	4	Nacht.3
hs1	26	*Er schaut sich das Zeichen an.*<br><br>"Wie alles sich zum Ganzen webt,<br>Eins in dem andern wirkt und lebt!<br>Wie Himmelskräfte auf und nieder steigen<br>Und sich die goldnen Eimer reichen!<br>Mit segenduftenden Schwingen<br>Vom Himmel durch die Erde dringen,<br>Harmonisch all das All durchklingen!	4	Nacht.3
hs1	27	Welch Schauspiel! Aber ach! ein Schauspiel nur!<br>Wo fass ich dich, unendliche Natur?<br>Euch Brüste, wo? Ihr Quellen alles Lebens,<br>An denen Himmel und Erde hängt,<br>Dahin die welke Brust sich drängt-<br>Ihr quellt, ihr tränkt, und schmacht ich so vergebens?"	4	Nacht.3

context marked in yellow

excerpt	stanza_nr	stanza_content	scene_nr	scene
hs1	28	*Er blättert unwillig weiter und erblickt das Zeichen des Erdgeistes.*<br><br>"Wie anders wirkt dies Zeichen auf mich ein!<br>Du, Geist der Erde, bist mir näher;<br>Schon fühl ich meine Kräfte höher,<br>Schon glüh ich wie von neuem Wein.<br>Ich fühle Mut, mich in die Welt zu wagen,<br>Der Erde Weh, der Erde Glück zu tragen,<br>Mit Stürmen mich herumzuschlagen<br>Und in des Schiffbruchs Knirschen nicht zu zagen.<br>Es wölkt sich über mir-<br>Der Mond verbirgt sein Licht-<br>Die Lampe schwindet!	5	Nacht.4
hs1	29	Es dampft! Es zucken rote Strahlen<br>Mir um das Haupt- Es weht<br>Ein Schauer vom Gewölb herab<br>Und faßt mich an!<br>Ich fühl's, du schwebst um mich, erflehter Geist<br>Enthülle dich!<br>Ha! wie's in meinem Herzen reißt!<br>Zu neuen Gefühlen<br>All meine Sinnen sich erwühlen!<br>Ich fühle ganz mein Herz dir hingegeben!<br>Du mußt! du mußt! und kostet es mein Leben!"	5	Nacht.4

context marked in yellow

excerpt	stanza_nr	stanza_content	scene_nr	scene
hs1	30	*Faust ergreift das Buch und spricht das Zeichen aus.*<br>*Eine rötliche Flamme zuckt, in der der Geist erscheint.*<br><br>"Wer ruft mir?" "Schreckliches Gesicht!"<br>"Du hast mich mächtig angezogen,<br>An meiner Sphäre lang gesogen,<br>Und nun-" "Weh! ich ertrag dich nicht!"	5	Nacht.4
hs1	31	"Du flehst, eratmend mich zu schauen,<br>Meine Stimme zu hören, mein Antlitz zu sehn;<br>Mich neigt dein mächtig Seelenflehn,<br>Da bin ich!- Welch erbärmlich Grauen<br>Faßt Übermenschen dich! Wo ist der Seele Ruf?<br>Wo ist die Brust, die eine Welt in sich erschuf<br>Und trug und hegte, die mit Freudebeben<br>Erschwoll, sich uns, den Geistern, gleich zu heben?	5	Nacht.4
hs1	32	Wo bist du, Faust, des Stimme mir erklang,<br>Der sich an mich mit allen Kräften drang?<br>Bist du es, der, von meinem Hauch umwittert,<br>In allen Lebenslagen zittert,<br>Ein furchtsam weggekrümmter Wurm?"	5	Nacht.4

context marked in yellow

excerpt	stanza_nr	stanza_content	scene_nr	scene
hs1	33	"Soll ich dir, Flammenbildung, weichen?<br>Ich bin's, bin Faust, bin deinesgleichen!"<br>"In Lebensfluten, im Tatensturm<br>Wall ich auf und ab,<br>Wehe hin und her!<br>Geburt und Grab,<br>Ein ewiges Meer,<br>Ein wechselndes Wehen,<br>Ein glühend Leben,<br>So schaff ich am laufenden Webstuhl der Zeit<br>Und wirke der Gottheit lebendiges Kleid."	5	Nacht.4
hs1	34	"Der du die weite Welt umschweifst,<br>Geschäftiger Geist, wie nah fühl ich mich dir!"<br>"Du gleichst dem Geist, den du begreifst,<br>Nicht mir!"<br>"Nicht dir?<br>Wem denn?<br>Ich Ebenbild der Gottheit!<br>Und nicht einmal dir!"	5	Nacht.4
hs1	35	*Der Geist ist wieder verschwunden.*<br>*Faust liegt am Boden. Es klopft.*<br><br>"O Tod! ich kenn's- das ist mein Famulus-<br>Es wird mein schönstes Glück zunichte!<br>Daß diese Fülle der Gesichte<br>Der trockne Schleicher stören muß!"	5	Nacht.4

context marked in yellow

excerpt	stanza_nr	stanza_content	scene_nr	scene
hs1	36	*Wagner tritt ein, im Schlafrock und mit Lampe in der Hand.*<br>*Faust wendet sich unwillig, als Wagner zu ihm spricht.*<br><br>Verzeiht! ich hör euch deklamieren;<br>Ihr last gewiß ein griechisch Trauerspiel?<br>In dieser Kunst möcht ich was profitieren,<br>Denn heutzutage wirkt das viel.<br>Ich hab es öfters rühmen hören,<br>Ein Komödiant könnt einen Pfarrer lehren.""	6	Nacht.5
hs1	37	"Ja, wenn der Pfarrer ein Komödiant ist;<br>Wie das denn wohl zuzeiten kommen mag."<br>"Ach! wenn man so in sein Museum gebannt ist,<br>Und sieht die Welt kaum einen Feiertag,<br>Kaum durch ein Fernglas, nur von weitem,<br>Wie soll man sie durch Überredung leiten?"	6	Nacht.5
hs1	38	"Wenn ihr's nicht fühlt, ihr werdet's nicht erjagen,<br>Wenn es nicht aus der Seele dringt<br>Und mit urkräftigem Behagen<br>Die Herzen aller Hörer zwingt.<br>Sitzt ihr nur immer! leimt zusammen,<br>Braut ein Ragout von andrer Schmaus<br>Und blast die kümmerlichen Flammen<br>Aus eurem Aschenhäufchen 'raus!<br>Bewundrung von Kindern und Affen,<br>Wenn euch darnach der Gaumen steht-<br>Doch werdet ihr nie Herz zu Herzen schaffen,<br>Wenn es euch nicht von Herzen geht."	6	Nacht.5

context marked in yellow

excerpt	stanza_nr	stanza_content	scene_nr	scene
hs2	39	*Im hochgewölbten, engen Zimmer.*<br>*Wagner unterhält sich mit Faust.*<br><br>"Allein der Vortrag macht des Redners Glück;<br>Ich fühl es wohl, noch bin ich weit zurück."<br>"Such Er den redlichen Gewinn!<br>Sei Er kein schellenlauter Tor!<br>Es trägt Verstand und rechter Sinn<br>Mit wenig Kunst sich selber vor!<br>Und wenn's euch Ernst ist, was zu sagen,<br>Ist's nötig, Worten nachzujagen?<br>Ja, eure Reden, die so blinkend sind,<br>In denen ihr der Menschheit Schnitzel kräuselt,<br>Sind unerquicklich wie der Nebelwind,<br>Der herbstlich durch die dürren Blätter säuselt!"	6	Nacht.5
hs2	40	"Ach Gott! die Kunst ist lang;<br>Und kurz ist unser Leben.<br>Mir wird, bei meinem kritischen Bestreben,<br>Doch oft um Kopf und Busen bang.<br>Wie schwer sind nicht die Mittel zu erwerben,<br>Durch die man zu den Quellen steigt!<br>Und eh man nur den halben Weg erreicht,<br>Muß wohl ein armer Teufel sterben."	6	Nacht.5
hs2	41	"Das Pergament, ist das der heil'ge Bronnen,<br>Woraus ein Trunk den Durst auf ewig stillt?<br>Erquickung hast du nicht gewonnen,<br>Wenn sie dir nicht aus eigner Seele quillt."	6	Nacht.5

context marked in yellow

excerpt	stanza_nr	stanza_content	scene_nr	scene
hs2	42	"Verzeiht! es ist ein groß Ergetzen,<br>Sich in den Geist der Zeiten zu versetzen;<br>Zu schauen, wie vor uns ein weiser Mann gedacht,<br>Und wie wir's dann zuletzt so herrlich weit gebracht."	6	Nacht.5
hs2	43	"O ja, bis an die Sterne weit!<br>Mein Freund, die Zeiten der Vergangenheit<br>Sind uns ein Buch mit sieben Siegeln.<br>Was ihr den Geist der Zeiten heißt,<br>Das ist im Grund der Herren eigner Geist,<br>In dem die Zeiten sich bespiegeln.<br>Da ist's denn wahrlich oft ein Jammer!<br>Man läuft euch bei dem ersten Blick davon.<br>Ein Kehrichtfaß und eine Rumpelkammer<br>Und höchstens eine Haupt- und Staatsaktion<br>Mit trefflichen pragmatischen Maximen,<br>Wie sie den Puppen wohl im Munde ziemen!"	6	Nacht.5
hs2	44	"Allein die Welt! des Menschen Herz und Geist!<br>Möcht jeglicher doch was davon erkennen."<br>"Ja, was man so erkennen heißt!<br>Wer darf das Kind beim Namen nennen?<br>Die wenigen, die was davon erkannt,<br>Die töricht g'nug ihr volles Herz nicht wahrten,<br>Dem Pöbel ihr Gefühl, ihr Schauen offenbarten,<br>Hat man von je gekreuzigt und verbrannt.<br>Ich bitt Euch, Freund, es ist tief in der Nacht,<br>Wir müssen's diesmal unterbrechen."	6	Nacht.5

context marked in yellow

excerpt	stanza_nr	stanza_content	scene_nr	scene
hs2	45	"Ich hätte gern nur immer fortgewacht,<br>Um so gelehrt mit Euch mich zu besprechen.<br>Doch morgen, als am ersten Ostertage,<br>Erlaubt mir ein' und andre Frage.<br>Mit Eifer hab' ich mich der Studien beflissen;<br>Zwar weiß ich viel, doch möcht' ich alles wissen."	6	Nacht.5
hs2	46	*Faust ist wieder allein.*<br><br>"Wie nur dem Kopf nicht alle Hoffnung schwindet,<br>Der immerfort an schalem Zeuge klebt,<br>Mit gier'ger Hand nach Schätzen gräbt,<br>Und froh ist, wenn er Regenwürmer findet!	6	Nacht.5
hs2	47	Darf eine solche Menschenstimme hier,<br>Wo Geisterfülle mich umgab, ertönen?<br>Doch ach! für diesmal dank ich dir,<br>Dem ärmlichsten von allen Erdensöhnen.<br>Du rissest mich von der Verzweiflung los,<br>Die mir die Sinne schon zerstören wollte.<br>Ach! die Erscheinung war so riesengroß,<br>Daß ich mich recht als Zwerg empfinden sollte.	7	Nacht.6

context marked in yellow

excerpt	stanza_nr	stanza_content	scene_nr	scene
hs2	48	Ich, Ebenbild der Gottheit, das sich schon<br>Ganz nah gedünkt dem Spiegel ew'ger Wahrheit,<br>Sein selbst genoß in Himmelsglanz und Klarheit,<br>Und abgestreift den Erdensohn;<br>Ich, mehr als Cherub, dessen freie Kraft<br>Schon durch die Adern der Natur zu fließen<br>Und, schaffend, Götterleben zu genießen<br>Sich ahnungsvoll vermaß, wie muß ich's büßen!<br>Ein Donnerwort hat mich hinweggerafft.	7	Nacht.6
hs2	49	Nicht darf ich dir zu gleichen mich vermessen;<br>Hab ich die Kraft dich anzuziehn besessen,<br>So hatt ich dich zu halten keine Kraft.<br>In jenem sel'gen Augenblicke<br>Ich fühlte mich so klein, so groß;<br>Du stießest grausam mich zurücke,<br>Ins ungewisse Menschenlos.<br>Wer lehret mich? was soll ich meiden?<br>Soll ich gehorchen jenem Drang?<br>Ach! unsre Taten selbst, so gut als unsre Leiden,<br>Sie hemmen unsres Lebens Gang.	7	Nacht.6
hs2	50	Dem Herrlichsten, was auch der Geist empfangen,<br>Drängt immer fremd und fremder Stoff sich an;<br>Wenn wir zum Guten dieser Welt gelangen,<br>Dann heißt das Beßre Trug und Wahn.<br>Die uns das Leben gaben, herrliche Gefühle<br>Erstarren in dem irdischen Gewühle.	7	Nacht.6

context marked in yellow

excerpt	stanza_nr	stanza_content	scene_nr	scene
hs2	51	Wenn Phantasie sich sonst mit kühnem Flug<br>Und hoffnungsvoll zum Ewigen erweitert,<br>So ist ein kleiner Raum ihr nun genug,<br>Wenn Glück auf Glück im Zeitenstrudel scheitert.<br>Die Sorge nistet gleich im tiefen Herzen,<br>Dort wirket sie geheime Schmerzen,<br>Unruhig wiegt sie sich und störet Lust und Ruh;<br>Sie deckt sich stets mit neuen Masken zu,<br>Sie mag als Haus und Hof, als Weib und Kind erscheinen,<br>Als Feuer, Wasser, Dolch und Gift;<br>Du bebst vor allem, was nicht trifft,<br>Und was du nie verlierst, das mußt du stets beweinen.	7	Nacht.6
hs2	52	Den Göttern gleich ich nicht! zu tief ist es gefühlt;<br>Dem Wurme gleich ich, der den Staub durchwühlt,<br>Den, wie er sich im Staube nährend lebt,<br>Des Wandrers Tritt vernichtet und begräbt.	7	Nacht.6
hs2	53	Ist es nicht Staub, was diese hohe Wand<br>Aus hundert Fächern mit verenget?<br>Der Trödel, der mit tausendfachem Tand<br>In dieser Mottenwelt mich dränget?<br>Hier soll ich finden, was mir fehlt?<br>Soll ich vielleicht in tausend Büchern lesen,<br>Daß überall die Menschen sich gequält,<br>Daß hie und da ein Glücklicher gewesen?-	7	Nacht.6

context marked in yellow

excerpt	stanza_nr	stanza_content	scene_nr	scene
hs2	54	Was grinsest du mir, hohler Schädel, her?<br>Als daß dein Hirn, wie meines, einst verwirret<br>Den leichten Tag gesucht und in der Dämmrung schwer,<br>Mit Lust nach Wahrheit, jämmerlich geirret.<br>Ihr Instrumente freilich spottet mein,<br>Mit Rad und Kämmen, Walz und Bügel:<br>Ich stand am Tor, ihr solltet Schlüssel sein;<br>Zwar euer Bart ist kraus, doch hebt ihr nicht die Riegel.	7	Nacht.6
hs2	55	Geheimnisvoll am lichten Tag<br>Läßt sich Natur des Schleiers nicht berauben,<br>Und was sie deinem Geist nicht offenbaren mag,<br>Das zwingst du ihr nicht ab mit Hebeln und mit Schrauben.<br>Du alt Geräte, das ich nicht gebraucht,<br>Du stehst nur hier, weil dich mein Vater brauchte.<br>Du alte Rolle, du wirst angeraucht,<br>Solang an diesem Pult die trübe Lampe schmauchte.	7	Nacht.6
hs2	56	Weit besser hätt ich doch mein Weniges verpraßt,<br>Als mit dem Wenigen belastet hier zu schwitzen!<br>Was du ererbt von deinen Vätern hast,<br>Erwirb es, um es zu besitzen.<br>Was man nicht nützt, ist eine schwere Last,<br>Nur was der Augenblick erschafft, das kann er nützen.	7	Nacht.6
hs2	57	Doch warum heftet sich mein Blick auf jene Stelle?<br>Ist jenes Fläschchen dort den Augen ein Magnet?<br>Warum wird mir auf einmal lieblich helle,<br>Als wenn im nächt'gen Wald uns Mondenglanz umweht?	8	Nacht.7.1

context marked in yellow

excerpt	stanza_nr	stanza_content	scene_nr	scene
hs2	58	Ich grüße dich, du einzige Phiole,<br>Die ich mit Andacht nun herunterhole!<br>In dir verehr ich Menschenwitz und Kunst.<br>Du Inbegriff der holden Schlummersäfte,<br>Du Auszug aller tödlich feinen Kräfte,<br>Erweise deinem Meister deine Gunst!<br>Ich sehe dich, es wird der Schmerz gelindert,<br>Ich fasse dich, das Streben wird gemindert,<br>Des Geistes Flutstrom ebbet nach und nach.<br>Ins hohe Meer werd ich hinausgewiesen,<br>Die Spiegelflut erglänzt zu meinen Füßen,<br>Zu neuen Ufern lockt ein neuer Tag.	8	Nacht.7.1
hs2	59	Ein Feuerwagen schwebt, auf leichten Schwingen,<br>An mich heran! Ich fühle mich bereit,<br>Auf neuer Bahn den Äther zu durchdringen,<br>Zu neuen Sphären reiner Tätigkeit.<br>Dies hohe Leben, diese Götterwonne!<br>Du, erst noch Wurm, und die verdienest du?<br>Ja, kehre nur der holden Erdensonne<br>Entschlossen deinen Rücken zu!	8	Nacht.7.1

context marked in yellow

excerpt	stanza_nr	stanza_content	scene_nr	scene
hs2	60	Vermesse dich, die Pforten aufzureißen,<br>Vor denen jeder gern vorüberschleicht!<br>Hier ist es Zeit, durch Taten zu beweisen,<br>Das Manneswürde nicht der Götterhöhe weicht,<br>Vor jener dunkeln Höhle nicht zu beben,<br>In der sich Phantasie zu eigner Qual verdammt,<br>Nach jenem Durchgang hinzustreben,<br>Um dessen engen Mund die ganze Hölle flammt;<br>Zu diesem Schritt sich heiter zu entschließen,<br>Und wär es mit Gefahr, ins Nichts dahin zu fließen.	8	Nacht.7.1
hs2	61	Nun komm herab, kristallne reine Schale!<br>Hervor aus deinem alten Futterale,<br>An die ich viele Jahre nicht gedacht!<br>Du glänzetst bei der Väter Freudenfeste,<br>Erheitertest die ernsten Gäste,<br>Wenn einer dich dem andern zugebracht.<br>Der vielen Bilder künstlich reiche Pracht,<br>Des Trinkers Pflicht, sie reimweis zu erklären,<br>Auf einen Zug die Höhlung auszuleeren,<br>Erinnert mich an manche Jugendnacht.	8	Nacht.7.1
hs2	62	Ich werde jetzt dich keinem Nachbar reichen,<br>Ich werde meinen Witz an deiner Kunst nicht zeigen.<br>Hier ist ein Saft, der eilig trunken macht;<br>Mit brauner Flut erfüllt er deine Höhle.<br>Den ich bereit, den ich wähle,<br>Der letzte Trunk sei nun, mit ganzer Seele,<br>Als festlich hoher Gruß, dem Morgen zugebracht!"	8	Nacht.7.1

context marked in yellow

excerpt	stanza_nr	stanza_content	scene_nr	scene
hs2	63	*Faust setzt die Schale an den Mund.*<br>*Plötzlich ertönt Glockenklang und Chorgesang. Der Chor singt, Faust reagiert.*<br><br>"Christ ist erstanden!<br>Freude dem Sterblichen,<br>Den die verderblichen,<br>Schleichenden, erblichen<br>Mängel unwanden."	9	Nacht.7.2
hs2	64	"Welch tiefes Summen, welch ein heller Ton,<br>Zieht mit Gewalt das Glas von meinem Munde?<br>Verkündiget ihr dumpfen Glocken schon<br>Des Osterfestes erste Feierstunde?<br>Ihr Chöre, singt ihr schon den tröstlichen Gesang?<br>Der einst, um Grabes Nacht, von Engelslippen klang,<br>Gewißheit einem neuen Bunde."	9	Nacht.7.2
hs2	65	"Mit Spezereien<br>Hatten wir ihn gepflegt,<br>Wir seine Treuen<br>Hatten ihn hingelegt;<br>Tücher und Binden<br>Reinlich unwanden wir,<br>Ach! und wir finden<br>Christ nicht mehr hier.	9	Nacht.7.2

context marked in yellow

excerpt	stanza_nr	stanza_content	scene_nr	scene
hs2	66	Christ ist erstanden!<br>Selig der Liebende,<br>Der die betrübende,<br>Heilsam und übende<br>Prüfung bestanden."	9	Nacht.7.2
hs2	67	"Was sucht ihr, mächtig und gelind,<br>Ihr Himmelstöne, mich am Staube?<br>Klingt dort umher, wo weiche Menschen sind.<br>Die Botschaft hör ich wohl, allein mir fehlt der Glaube;<br>Das Wunder ist des Glaubens liebstes Kind.<br>Zu jenen Sphären wag ich nicht zu streben,<br>Woher die holde Nachricht tönt;<br>Und doch, an diesen Klang von Jugend auf gewöhnt,<br>Ruft er auch jetzt zurück mich in das Leben.	9	Nacht.7.2
hs2	68	Sonst stürzte sich der Himmelsliebe Kuß<br>Auf mich herab in ernster Sabbatstille;<br>Da klang so ahnungsvoll des Glockentones Fülle,<br>Und ein Gebet war brünstiger Genuß;<br>Ein unbegreiflich holdes Sehnen<br>Trieb mich, durch Wald und Wiesen hinzugehn,<br>Und unter tausend heißen Tränen<br>Fühlt ich mir eine Welt entstehn.	9	Nacht.7.2

context marked in yellow

excerpt	stanza_nr	stanza_content	scene_nr	scene
hs2	69	Dies Lied verkündete der Jugend muntre Spiele,<br>Der Frühlingsfeier freies Glück;<br>Erinnrung hält mich nun, mit kindlichem Gefühle,<br>Vom letzten, ernsten Schritt zurück.<br>O tönet fort, ihr süßen Himmelslieder!<br>Die Träne quillt, die Erde hat mich wieder!"	9	Nacht.7.2
hs2	70	"Hat der Begrabene<br>Schon sich nach oben,<br>Lebend Erhabene,<br>Herrlich erhoben;<br>Ist er in Werdeluft<br>Schaffender Freude nah:<br>Ach! an der Erde Brust<br>Sind wir zum Leide da.<br>Ließ er die Seinen<br>Schmachtend uns hier zurück;<br>Ach! wir beweinen,<br>Meister, dein Glück!	9	Nacht.7.2

context marked in yellow

excerpt	stanza_nr	stanza_content	scene_nr	scene
hs2	71	Christ ist erstanden,<br>Aus der Verwesung Schoß.<br>Reißet von Banden<br>Freudig euch los!<br>Tätig ihn preisenden,<br>Liebe beweisenden,<br>Brüderlich speisenden,<br>Predigend reisenden,<br>Wonne verheißenden<br>Euch ist der Meister nah,<br>Euch ist er da!"	9	Nacht.7.2
mg1	1	*Auf der Straße.*<br>*Faust unterhält sich mit Mephistopheles.*<br><br>"Wie ist's? Will's fördern? Will's bald gehn?"<br>"Ah bravo! Find ich Euch in Feuer?<br>In kurzer Zeit ist Gretchen Euer.<br>Heut abend sollt Ihr sie bei Nachbar' Marthen sehn:<br>Das ist ein Weib wie auserlesen<br>Zum Kuppler- und Zigeunerwesen!"<br>"So recht!" "Doch wird auch was von uns begehrt."<br>"Ein Dienst ist wohl des andern wert."	10	Straße.2

context marked in yellow

excerpt	stanza_nr	stanza_content	scene_nr	scene
mg1	2	"Wir legen nur ein gültig Zeugnis nieder,<br>Daß ihres Ehherrn ausgereckte Glieder<br>In Padua an heil'ger Stätte ruhn."<br>"Sehr klug! Wir werden erst die Reise machen müssen!"<br>"Sancta Simplicitas! darum ist's nicht zu tun;<br>Bezeugt nur, ohne viel zu wissen."<br>"Wenn Er nichts Bessers hat, so ist der Plan zerrissen."	10	Straße.2
mg1	3	"O heil'ger Mann! Da wärt Ihr's nun!<br>Ist es das erstemal in eurem Leben,<br>Daß Ihr falsch Zeugnis abgelegt?<br>Habt Ihr von Gott, der Welt und was sich drin bewegt,<br>Vom Menschen, was sich ihm in den Kopf und Herzen regt,<br>Definitionen nicht mit großer Kraft gegeben?<br>Mit frecher Stirne, kühner Brust?<br>Und wollt Ihr recht ins Innre gehen,<br>Habt Ihr davon, Ihr müßt es grad gestehen,<br>So viel als von Herrn Schwerdtleins Tod gewußt!"	10	Straße.2
mg1	4	"Du bist und bleibst ein Lügner, ein Sophiste."<br>"Ja, wenn man's nicht ein bißchen tiefer wüßte.<br>Denn morgen wirst, in allen Ehren,<br>Das arme Gretchen nicht betören<br>Und alle Seelenlieb ihr schwören?"<br>"Und zwar von Herzen." "Gut und schön!<br>Dann wird von ewiger Treu und Liebe,<br>von einzig überallmächt'gem Triebe -<br>Wird das auch so von Herzen gehn?"	10	Straße.2

context marked in yellow

excerpt	stanza_nr	stanza_content	scene_nr	scene
mg1	5	"Laß das! Es wird!- Wenn ich empfinde,<br>Für das Gefühl, für das Gewühl<br>Nach Namen suche, keinen finde,<br>Dann durch die Welt mit allen Sinnen schweife,<br>Nach allen höchsten Worten greife,<br>Und diese Glut, von der ich brenne,<br>Unendlich, ewig, ewig nenne,<br>Ist das ein teuflisch Lügenspiel?"	10	Straße.2
mg1	6	"Ich hab doch recht!" "Hör! merk dir dies -<br>Ich bitte dich, und schone meine Lunge -:<br>Wer recht behalten will und hat nur eine Zunge,<br>Behält's gewiß.<br>Und komm, ich hab des Schwätzens Überdruß,<br>Denn du hast recht, vorzüglich weil ich muß."	10	Straße.2
mg1	7	*Garten. Marthe und Mephistopheles im Hintergrund.*<br>*Gretchen am Arm von Faust, spricht mit ihm.*<br><br>"Ich fühl es wohl, daß mich der Herr nur schont,<br>Herab sich läßt, mich zu beschämen.<br>Ein Reisender ist so gewohnt,<br>Aus Gütigkeit fürliebzunehmen;<br>Ich weiß zu gut, daß solch erfahrnen Mann<br>Mein arm Gespräch nicht unterhalten kann."	11	Garten.1

context marked in yellow

excerpt	stanza_nr	stanza_content	scene_nr	scene
mg1	8	*Faust küsst die Hand von Gretchen.*<br><br>"Ein Blick von dir, ein Wort mehr unterhält<br>Als alle Weisheit dieser Welt."<br>"Inkommodiert Euch nicht! Wie könnt Ihr sie nur küssen?<br>Sie ist so garstig, ist so rauh!<br>Was hab ich nicht schon alles schaffen müssen!<br>Die Mutter ist gar zu genau."	11	Garten.1
mg1	9	*Marthe und Mephistopheles spazieren vorbei.*<br>*Die zwei unterhalten sich miteinander, Marthe spricht zuerst.*<br><br>"Und Ihr, mein Herr, Ihr reist so immer fort?"<br>"Ach, daß Gewerb und Pflicht uns dazu treiben!<br>Mit wieviel Schmerz verläßt man manchen Ort<br>Und darf doch nun einmal nicht bleiben!"	11	Garten.1
mg1	10	"In raschen Jahren geht's wohl an<br>So um und um frei durch die Welt zu streifen;<br>Doch kömmt die böse Zeit heran,<br>Und sich als Hagestolz allein zum Grab zu schleifen,<br>Das hat noch keinem wohlgetan."<br>"Mit Grausen seh ich das von weiten."<br>"Drum, werter Herr, beratet Euch in Zeiten."	11	Garten.1

context marked in yellow

excerpt	stanza_nr	stanza_content	scene_nr	scene
mg1	11	*Wieder Gretchen und Faust.*<br><br>"Ja, aus den Augen, aus dem Sinn!<br>Die Höflichkeit ist Euch geläufig;<br>Allein Ihr habt der Freunde häufig,<br>Sie sind verständiger, als ich bin."	11	Garten.1
mg1	12	"O Beste! glaube, was man so verständig nennt,<br>Ist oft mehr Eitelkeit und Kurzsinn." "Wie?"<br>"Ach, daß die Einfalt, daß die Unschuld nie<br>Sich selbst und ihren heil'gen Wert erkennt!<br>Daß Demut Niedrigkeit, die höchsten Gaben<br>Der liebevoll austeilenden Natur-"<br>"Denkt Ihr an mich ein Augenblickchen nur,<br>Ich werde Zeit genug an Euch zu denken haben."	11	Garten.1
mg1	13	"Ihr seid wohl viel allein?"<br>"Ja, unsre Wirtschaft ist nur klein,<br>Und doch will sie versehen sein.<br>Wir haben keine Magd; muß kochen, fegen, stricken<br>Und nähn und laufen früh und spat;<br>Und meine Mutter ist in allen Stücken<br>So akkurat!<br>Nicht daß sie just so sehr sich einzuschränken hat;<br>Wir könnten uns weit eh'r als andre regen:<br>Mein Vater hinterließ ein hübsch Vermögen,<br>Ein Häuschen und ein Gärtchen vor der Stadt.	11	Garten.1

context marked in yellow

excerpt	stanza_nr	stanza_content	scene_nr	scene
mg1	14	Doch hab ich jetzt so ziemlich stille Tage:<br>Mein Bruder ist Soldat,<br>Mein Schwesterchen ist tot.<br>Ich hatte mit dem Kind wohl meine liebe Not;<br>Doch übernähm ich gern noch einmal alle Plage,<br>So lieb war mir das Kind."	11	Garten.1
mg1	15	"Ein Engel, wenn dir's glich."<br>"Ich zog es auf, und herzlich liebt es mich.<br>Es war nach meines Vaters Tod geboren.<br>Die Mutter gaben wir verloren,<br>So elend wie sie damals lag,<br>Und sie erholte sich sehr langsam, nach und nach.<br>Da konnte sie nun nicht dran denken,<br>Das arme Würmchen selbst zu tränken,<br>Und so erzog ich's ganz allein,<br>Mit Milch und Wasser, so ward's mein<br>Auf meinem Arm, in meinem Schoß<br>War's freundlich, zappelte, ward groß."	11	Garten.1
mg1	16	"Du hast gewiß das reinste Glück empfunden."<br>"Doch auch gewiß gar manche schwere Stunden.<br>Des Kleinen Wiege stand zu Nacht<br>An meinem Bett; es durfte kaum sich regen,<br>War ich erwacht;<br>Bald mußt ich's tränken, bald es zu mir legen<br>Bald, wenn's nicht schwieg, vom Bett aufstehn<br>Und tänzelnd in der Kammer auf und nieder gehn,<br>Und früh am Tage schon am Waschtrog stehn;	11	Garten.1

context marked in yellow

excerpt	stanza_nr	stanza_content	scene_nr	scene
mg1	17	Dann auf dem Markt und an dem Herde sorgen,<br>Und immer fort wie heut so morgen.<br>Da geht's, mein Herr, nicht immer mutig zu;<br>Doch schmeckt dafür das Essen, schmeckt die Ruh."	11	Garten.1
mg1	18	*Wieder Marthe und Mephistopheles.*<br><br>"Die armen Weiber sind doch übel dran:<br>Ein Hagestolz ist schwerlich zu bekehren."<br>"Es käme nur auf Euresgleichen an,<br>Mich eines Bessern zu belehren."<br>"Sagt grad, mein Herr, habt Ihr noch nichts gefunden?<br>Hat sich das Herz nicht irgendwo gebunden?"<br>"Das Sprichwort sagt: Ein eigner Herd,<br>Ein braves Weib sind Gold und Perlen wert."	11	Garten.1
mg1	19	"Ich meine: ob Ihr niemals Lust bekommen?"<br>"Man hat mich überall recht höflich aufgenommen."<br>"Ich wollte sagen: ward's nie Ernst in Eurem Herzen?"<br>"Mit Frauen soll man sich nie unterstehn zu scherzen."<br>"Ach, Ihr versteht mich nicht!" "Das tut mir herzlich leid!<br>Doch ich versteh- daß Ihr sehr gütig seid."	11	Garten.1

context marked in yellow

excerpt	stanza_nr	stanza_content	scene_nr	scene
mg1	20	*Faust und Gretchen.*<br><br>"Du kanntest mich, o kleiner Engel, wieder,<br>Gleich als ich in den Garten kam?"<br>"Saht Ihr es nicht, ich schlug die Augen nieder."<br>"Und du verzeihst die Freiheit, die ich nahm?<br>Was sich die Frechheit unterfangen,<br>Als du jüngst aus dem Dom gegangen?"	11	Garten.1
mg1	21	"Ich war bestürzt, mir war das nie geschehn;<br>Es konnte niemand von mir Übels sagen.<br>Ach, dacht ich, hat er in deinem Betragen<br>Was Freches, Unanständiges gesehn?<br>Es schien ihn gleich nur anzuwandeln,<br>Mit dieser Dirne gradehin zu handeln.<br>Gesteh ich's doch! Ich wußte nicht, was sich<br>Zu Eurem Vorteil hier zu regen gleich begonnte;<br>Allein gewiß, ich war recht bös auf mich,<br>Daß ich auf Euch nicht böser werden konnte."	11	Garten.1

context marked in yellow

excerpt	stanza_nr	stanza_content	scene_nr	scene
mg1	22	*Gretchen nimmt sich eine Blume und zupft nacheinander die einzelnen Blätter ab.*<br>*Faust wendet sich ihr zu.*<br><br>"Süß Liebchen!" "Laßt einmal!" "Was soll das? Einen Strauß?"<br>"Nein, es soll nur ein Spiel." "Wie?" "Geht! Ihr lacht mich aus."<br>"Was murmelst du?" "Er liebt mich - liebt mich nicht."<br>"Du holdes Himmelsangesicht!"<br>"Liebt mich- nicht- liebt mich- nicht-<br>Er liebt mich!"<br>"Ja, mein Kind! Laß dieses Blumenwort<br>Dir Götterausspruch sein. Er liebt dich!<br>Verstehst du, was das heißt? Er liebt dich!"<br>"Mich überläuft's!"	11	Garten.1
mg1	23	"O schaudre nicht! Laß diesen Blick,<br>Laß diesen Händedruck dir sagen<br>Was unaussprechlich ist:<br>Sich hinzugeben ganz und eine Wonne<br>Zu fühlen, die ewig sein muß!<br>Ewig!- Ihr Ende würde Verzweiflung sein<br>Nein, kein Ende! Kein Ende!"	11	Garten.1

context marked in yellow

excerpt	stanza_nr	stanza_content	scene_nr	scene
mg1	24	*Gretchen drückt Faust die Hände, dann laufen beide nacheinander weg.*<br>*Marthe und Mephistopheles kommen hinzu, unterhalten sich.*<br><br>"Die Nacht bricht an." "Ja, und wir wollen fort."<br>"Ich bät Euch, länger hier zu bleiben,<br>Allein es ist ein gar zu böser Ort.<br>Es ist, als hätte niemand nichts zu treiben<br>Und nichts zu schaffen,<br>Als auf des Nachbarn Schritt und Tritt zu gaffen,<br>Und man kommt ins Gered, wie man sich immer stellt.<br>Und unser Pärchen?" "Ist den Gang dort aufgeflogen.<br>Mutwill'ge Sommervögel!" "Er scheint ihr gewogen.<br>Und sie ihm auch. Das ist der Lauf der Welt."	11	Garten.1
mg1	25	*Gretchen springt in ein Gartenhaus und versteckt sich,*<br>*Faust folgt ihr und sie küssen sich.*<br>*Später kommen Mephistopheles und Marthe hinzu und klopfen an.*<br><br>[Gretchen und Faust]<br>"Er kommt!" "Ach, Schelm, so neckst du mich! Treff' ich dich!"<br>"Bester Mann! von Herzen lieb ich dich!"<br>[Es klopft, alle unterhalten sich durch die Tür.]<br>"Wer da?" "Gut Freund!" "Ein Tier!""Es ist wohl Zeit zu scheiden."<br>[wieder Gretchen und Faust]<br>"Ja, es ist spät, mein Herr." "Darf ich Euch nicht geleiten?"<br>"Die Mutter würde mich- Lebt wohl!" "Muß ich denn gehn?<br>Lebt wohl!" "Ade!" "Auf baldig Wiedersehn!"	12	Garten.2

context marked in yellow

excerpt	stanza_nr	stanza_content	scene_nr	scene
mg1	26	*Gretchen wieder alleine.*<br><br>"Du lieber Gott! was so ein Mann<br>Nicht alles, alles denken kann!<br>Beschämt nur steh ich vor ihm da<br>Und sag zu allen Sachen ja.<br>Bin doch ein arm unwissend Kind,<br>Begreife nicht, was er an mir findt."	12	Garten.2
mg1	27	*Im Wald.*<br>*Faust ist alleine.*<br><br>"Erhabner Geist, du gabst mir, gabst mir alles,<br>Warum ich bat. Du hast mir nicht umsonst<br>Dein Angesicht im Feuer zugewendet.<br>Gabst mir die herrliche Natur zum Königreich,<br>Kraft, sie zu fühlen, zu genießen. Nicht<br>Kalt staunenden Besuch erlaubst du nur,<br>Vergönnest mir, in ihre tiefe Brust<br>Wie in den Busen eines Freunds zu schauen.<br>Du führst die Reihe der Lebendigen<br>Vor mir vorbei und lehrst mich meine Brüder<br>Im stillen Busch, in Luft und Wasser kennen.	13	Wald.1

context marked in yellow

excerpt	stanza_nr	stanza_content	scene_nr	scene
mg1	28	Und wenn der Sturm im Walde braust und knarrt,<br>Die Riesenfichte stürzend Nachbaräste<br>Und Nachbarstämme quetschend niederstreift<br>Und ihrem Fall dumpf hohl der Hügel donnert,<br>Dann führst du mich zur sichern Höhle, zeigst<br>Mich dann mir selbst, und meiner eignen Brust<br>Geheime tiefe Wunder öffnen sich.<br>Und steigt vor meinem Blick der reine Mond<br>Besänftigend herüber, schweben mir<br>Von Felsenwänden, aus dem feuchten Busch<br>Der Vorwelt silberne Gestalten auf<br>Und lindern der Betrachtung strenge Lust.	13	Wald.1
mg1	29	O daß dem Menschen nichts Vollkommnes wird,<br>Empfind ich nun. Du gabst zu dieser Wonne,<br>Die mich den Göttern nah und näher bringt,<br>Mir den Gefährten, den ich schon nicht mehr<br>Entbehren kann, wenn er gleich, kalt und frech,<br>Mich vor mir selbst erniedrigt und zu Nichts,<br>Mit einem Worthauch, deine Gaben wandelt.<br>Er facht in meiner Brust ein wildes Feuer<br>Nach jenem schönen Bild geschäftig an.<br>So tauml ich von Begierde zu Genuß,<br>Und im Genuß verschmacht ich nach Begierde."	14	Wald.2

context marked in yellow

excerpt	stanza_nr	stanza_content	scene_nr	scene
mg1	30	*Mephistopheles tritt auf und ergreift das Wort.*<br><br>"Habt Ihr nun bald das Leben gnug geführt?<br>Wie kann's Euch in die Länge freuen?<br>Es ist wohl gut, daß man's einmal probiert<br>Dann aber wieder zu was Neuen!"	15	Wald.3
mg1	31	"Ich wollt, du hättest mehr zu tun,<br>Als mich am guten Tag zu plagen."<br>"Nun, nun! ich laß dich gerne ruhn,<br>Du darfst mir's nicht im Ernste sagen.<br>An dir Gesellen, unhold, barsch und toll,<br>Ist wahrlich wenig zu verlieren.<br>Den ganzen Tag hat man die Hände voll!<br>Was ihm gefällt und was man lassen soll,<br>Kann man dem Herrn nie an der Nase spüren."	15	Wald.3
mg1	32	"Das ist so just der rechte Ton!<br>Er will noch Dank, daß er mich ennuyiert."<br>"Wie hättst du, armer Erdensohn<br>Dein Leben ohne mich geführt?<br>Vom Kribskrabs der Imagination<br>Hab ich dich doch auf Zeiten lang kuriert;<br>Und wär ich nicht, so wärst du schon<br>Von diesem Erdball abspaziert.	16	Wald.4

context marked in yellow

excerpt	stanza_nr	stanza_content	scene_nr	scene
mg1	33	Was hast du da in Höhlen, Felsenritzen<br>Dich wie ein Schuhu zu versitzen?<br>Was schlurfst aus dumpfem Moos und triefendem Gestein<br>Wie eine Kröte Nahrung ein?<br>Ein schöner, süßer Zeitvertreib!<br>Dir steckt der Doktor noch im Leib."	16	Wald.4
mg2	34	*Faust und Mephistopheles im Wald.*<br>*Faust ergreift das Wort.*<br><br>"Verstehst du, was für neue Lebenskraft<br>Mir dieser Wandel in der Öde schafft?<br>Ja, würdest du es ahnen können,<br>Du wärest Teufel gnug, mein Glück mir nicht zu gönnen."	16	Wald.4
mg2	35	"Ein überirdisches Vergnügen.<br>In Nacht und Tau auf den Gebirgen liegen<br>Und Erd und Himmel wonniglich umfassen,<br>Zu einer Gottheit sich aufschwellen lassen,<br>Der Erde Mark mit Ahnungsdrang durchwühlen,<br>Alle sechs Tagewerk im Busen fühlen,<br>In stolzer Kraft ich weiß nicht was genießen,<br>Bald liebewonniglich in alles überfließen,<br>Verschwunden ganz der Erdensohn,<br>Und dann die hohe Intuition -	16	Wald.4

context marked in yellow

excerpt	stanza_nr	stanza_content	scene_nr	scene
mg2	36	Ich darf nicht sagen, wie - zu schließen."<br>"Pfui über dich!" "Das will Euch nicht behagen;<br>Ihr habt das Recht, gesittet pfui zu sagen.<br>Man darf das nicht vor keuschen Ohren nennen,<br>Was keusche Herzen nicht entbehren können.<br>Und kurz und gut, ich gönn Ihm das Vergnügen,<br>Gelegentlich sich etwas vorzulügen;<br>Doch lange hält Er das nicht aus.<br>Du bist schon wieder abgetrieben<br>Und, währt es länger, aufgerieben<br>In Tollheit oder Angst und Graus.	16	Wald.4
mg2	37	Genug damit! Dein Liebchen sitzt dadrinne,<br>Und alles wird ihr eng und trüb.<br>Du kommst ihr gar nicht aus dem Sinne,<br>Sie hat dich übermächtig lieb.<br>Erst kam deine Liebeswut übergeflossen,<br>Wie vom geschmolznen Schnee ein Bächlein übersteigt;<br>Du hast sie ihr ins Herz gegossen,<br>Nun ist dein Bächlein wieder seicht.	17	Wald.5
mg2	38	Mich dünkt, anstatt in Wäldern zu thronen,<br>Ließ' es dem großen Herren gut,<br>Das arme affenjunge Blut<br>Für seine Liebe zu belohnen.	17	Wald.5

context marked in yellow

excerpt	stanza_nr	stanza_content	scene_nr	scene
mg2	39	Die Zeit wird ihr erbärmlich lang;<br>Sie steht am Fenster, sieht die Wolken ziehn<br>Über die alte Stadtmauer hin.<br>Wenn ich ein Vöglein wär! so geht ihr Gesang<br>Tage lang, halbe Nächte lang.<br>Einmal ist sie munter, meist betrübt,<br>Einmal recht ausgeweint,<br>Dann wieder ruhig, wie's scheint,<br>Und immer verliebt."	17	Wald.5
mg2	40	"Schlange! Schlange!"<br>"Gelt! daß ich dich fange!"<br>"Verruchter! hebe dich von hinnen,<br>Und nenne nicht das schöne Weib!<br>Bring die Begier zu ihrem süßen Leib<br>Nicht wieder vor die halb verrückten Sinnen!"<br>"Was soll es denn? Sie meint, du seist entflohn,<br>Und halb und halb bist du es schon."	17	Wald.5
mg2	41	"Ich bin ihr nah, und wär ich noch so fern,<br>Ich kann sie nie vergessen, nie verlieren<br>Ja, ich beneide schon den Leib des Herrn,<br>Wenn ihre Lippen ihn indes berühren."<br>"Gar wohl, mein Freund! Ich hab Euch oft beneidet<br>Ums Zwillingspaar, das unter Rosen weidet."	17	Wald.5

context marked in yellow

excerpt	stanza_nr	stanza_content	scene_nr	scene
mg2	42	"Entfliehe, Kuppler!" "Schön! Ihr schimpft, und ich muß lachen.<br>Der Gott, der Bub' und Mädchen schuf,<br>Erkannte gleich den edelsten Beruf,<br>Auch selbst Gelegenheit zu machen.<br>Nur fort, es ist ein großer Jammer!<br>Ihr sollt in Eures Liebchens Kammer,<br>Nicht etwa in den Tod."	17	Wald.5
mg2	43	"Was ist die Himmelsfreud in ihren Armen?<br>Laß mich an ihrer Brust erwarmen!<br>Fühl ich nicht immer ihre Not?<br>Bin ich der Flüchtling nicht? der Unbehauste?<br>Der Unmensch ohne Zweck und Ruh,<br>Der wie ein Wassersturz von Fels zu Felsen brauste,<br>Begierig wütend nach dem Abgrund zu?<br>Und seitwärts sie, mit kindlich dumpfen Sinnen,<br>Im Hüttchen auf dem kleinen Alpenfeld,<br>Und all ihr häusliches Beginnen<br>Umfangen in der kleinen Welt.	18	Wald.6

context marked in yellow

excerpt	stanza_nr	stanza_content	scene_nr	scene
mg2	44	Und ich, der Gottverhaßte,<br>Hatte nicht genug,<br>Daß ich die Felsen faßte<br>Und sie zu Trümmern schlug!<br>Sie, ihren Frieden mußt ich untergraben!<br>Du, Hölle, mußtest dieses Opfer haben.<br>Hilf, Teufel, mir die Zeit der Angst verkürzen.<br>Was muß geschehn, mag's gleich geschehn!<br>Mag ihr Geschick auf mich zusammenstürzen<br>Und sie mit mir zugrunde gehn!"	18	Wald.6
mg2	45	"Wie's wieder siedet, wieder glüht!<br>Geh ein und tröste sie, du Tor!<br>Wo so ein Köpfchen keinen Ausgang sieht,<br>Stellt er sich gleich das Ende vor.<br>Es lebe, wer sich tapfer hält!<br>Du bist doch sonst so ziemlich eingeteufelt.<br>Nichts Abgeschmackters find ich auf der Welt<br>Als einen Teufel, der verzweifelt."	19	Wald.7
mg2	46	*Gretchen, alleine am Spinnrad in ihrer Stube.*<br><br>"Meine Ruh ist hin,<br>Mein Herz ist schwer;<br>Ich finde sie nimmer<br>und nimmermehr.	20	Gretchens

context marked in yellow

excerpt	stanza_nr	stanza_content	scene_nr	scene
mg2	47	Wo ich ihn nicht hab,<br>Ist mir das Grab,<br>Die ganze Welt<br>Ist mir vergällt.	20	Gretchens
mg2	48	Mein armer Kopf<br>Ist mir verrückt,<br>Meiner armer Sinn<br>Ist mir zerstückt.	20	Gretchens
mg2	49	Meine Ruh ist hin,<br>Mein Herz ist schwer,<br>Ich finde sie nimmer<br>und nimmermehr.	20	Gretchens
mg2	50	Nach ihm nur schau ich<br>Zum Fenster hinaus,<br>Nach ihm nur geh ich<br>Aus dem Haus.	20	Gretchens
mg2	51	Sein hoher Gang,<br>Sein edle Gestalt,<br>Seines Mundes Lächeln,<br>Seiner Augen Gewalt,	20	Gretchens
mg2	52	Und seiner Rede<br>Zauberfluß,<br>Sein Händedruck,<br>Und ach! sein Kuß!	20	Gretchens

context marked in yellow

excerpt	stanza_nr	stanza_content	scene_nr	scene
mg2	53	Meine Ruh ist hin,<br>Mein Herz ist schwer,<br>Ich finde sie nimmer<br>und nimmermehr.	20	Gretchens
mg2	54	Mein Busen drängt<br>Sich nach ihm hin,<br>Ach dürft ich fassen<br>Und halten ihn,<br>Und küssen ihn,<br>So wie ich wollt,<br>An seinen Küssen<br>Vergehen sollt!"	20	Gretchens
mg2	55	*Wieder im Garten.*<br>*Gretchen spricht zu Faust.*<br><br>"Versprich mir, Heinrich!" "Was ich kann!"<br>"Nun sag, wie hast du's mit der Religion?<br>Du bist ein herzlich guter Mann,<br>Allein ich glaub, du hältst nicht viel davon."<br>"Laß das, mein Kind! Du fühlst, ich bin dir gut;<br>Für meine Lieben ließ' ich Leib und Blut,<br>Will niemand sein Gefühl und seine Kirche rauben."	21	Garten.3.1

context marked in yellow

excerpt	stanza_nr	stanza_content	scene_nr	scene
mg2	56	"Das ist nicht recht, man muß dran glauben."<br>"Muß man?" "Ach! wenn ich etwas auf dich konnte!<br>Du ehrst auch nicht die heil'gen Sakramente."<br>"Ich ehre sie." "Doch ohne Verlangen.<br>Zur Messe, zur Beichte bist du lange nicht gegangen.<br>Glaubst du an Gott?" "Mein Liebchen, wer darf sagen:<br>Ich glaub an Gott?<br>Magst Priester oder Weise fragen,<br>Und ihre Antwort scheint nur Spott<br>Über den Frager zu sein." "So glaubst du nicht?"	22	Garten.3.2
mg2	56	"Das ist nicht recht, man muß dran glauben."<br>"Muß man?" "Ach! wenn ich etwas auf dich konnte!<br>Du ehrst auch nicht die heil'gen Sakramente."<br>"Ich ehre sie." "Doch ohne Verlangen.<br>Zur Messe, zur Beichte bist du lange nicht gegangen.<br>Glaubst du an Gott?" "Mein Liebchen, wer darf sagen:<br>Ich glaub an Gott?<br>Magst Priester oder Weise fragen,<br>Und ihre Antwort scheint nur Spott<br>Über den Frager zu sein." "So glaubst du nicht?"	23	Garten.3.3
mg2	57	"Mißhör mich nicht, du holdes Angesicht!<br>Wer darf ihn nennen?<br>Und wer bekennen:<br>Ich glaub ihn!?<br>Wer empfinden,<br>Und sich unterwinden<br>Zu sagen: 'Ich glaub ihn nicht!'?	23	Garten.3.3

context marked in yellow

excerpt	stanza_nr	stanza_content	scene_nr	scene
mg2	58	Der Allumfasser,<br>Der Allerhalter,<br>Faßt und erhält er nicht<br>Dich, mich, sich selbst?<br>Wölbt sich der Himmel nicht da droben?<br>Liegt die Erde nicht hier unten fest?	23	Garten.3.3
mg2	59	Und steigen freundlich blickend<br>Ewige Sterne nicht herauf?<br>Schau ich nicht Aug in Auge dir,<br>Und drängt nicht alles<br>Nach Haupt und Herzen dir,<br>Und webt in ewigem Geheimnis<br>Unsichtbar sichtbar neben dir?	23	Garten.3.3
mg2	60	Erfüll davon dein Herz, so groß es ist,<br>Und wenn du ganz in dem Gefühle selig bist,<br>Nenn es dann, wie du willst,<br>Nenn's Glück! Herz! Liebe! Gott<br>Ich habe keinen Namen<br>Dafür! Gefühl ist alles;<br>Name ist Schall und Rauch,<br>Umnebelnd Himmelsglut."	23	Garten.3.3

context marked in yellow

excerpt	stanza_nr	stanza_content	scene_nr	scene
mg2	61	"Das ist alles recht schön und gut;<br>Ungefähr sagt das der Pfarrer auch,<br>Nur mit ein bißchen andern Worten."<br>"Es sagen's allerorten<br>Alle Herzen unter dem himmlischen Tage,<br>Jedes in seiner Sprache;<br>Warum nicht ich in der meinen?"	23	Garten.3.3
mg2	62	"Wenn man's so hört, möcht's leidlich scheinen,<br>Steht aber doch immer schief darum;<br>Denn du hast kein Christentum."<br>"Liebs Kind!" "Es tut mir lange schon weh,<br>Daß ich dich in der Gesellschaft seh."<br>"Wieso?" "Der Mensch, den du da bei dir hast,<br>Ist mir in tiefer innrer Seele verhaßt;<br>Es hat mir in meinem Leben<br>So nichts einen Stich ins Herz gegeben<br>Als des Menschen widrig Gesicht."	24	Garten.3.4
mg2	62	"Wenn man's so hört, möcht's leidlich scheinen,<br>Steht aber doch immer schief darum;<br>Denn du hast kein Christentum."<br>"Liebs Kind!" "Es tut mir lange schon weh,<br>Daß ich dich in der Gesellschaft seh."<br>"Wieso?" "Der Mensch, den du da bei dir hast,<br>Ist mir in tiefer innrer Seele verhaßt;<br>Es hat mir in meinem Leben<br>So nichts einen Stich ins Herz gegeben<br>Als des Menschen widrig Gesicht."	25	Garten.3.5

context marked in yellow

excerpt	stanza_nr	stanza_content	scene_nr	scene
mg2	63	"Liebe Puppe, fürcht ihn nicht!"<br>"Seine Gegenwart bewegt mir das Blut.<br>Ich bin sonst allen Menschen gut;<br>Aber wie ich mich sehne, dich zu schauen,<br>Hab ich vor dem Menschen ein heimlich Grauen,<br>Und halt ihn für einen Schelm dazu!<br>Gott verzeih mir's, wenn ich ihm unrecht tu!"	25	Garten.3.5
mg2	64	"Es muß auch solche Käuze geben."<br>"Wollte nicht mit seinesgleichen leben!<br>Kommt er einmal zur Tür herein,<br>Sieht er immer so spöttisch drein<br>Und halb ergrimmt;<br>Man sieht, daß er an nichts keinen Anteil nimmt;<br>Es steht ihm an der Stirn geschrieben,<br>Daß er nicht mag eine Seele lieben.<br>Mir wird's so wohl in deinem Arm,<br>So frei, so hingegeben warm,<br>Und seine Gegenwart schnürt mir das Innre zu."<br>"Du ahnungsvoller Engel du!"	25	Garten.3.5
mg2	65	"Das übermannt mich so sehr,<br>Daß, wo er nur mag zu uns treten,<br>Mein ich sogar, ich liebte dich nicht mehr.<br>Auch, wenn er da ist, könnt ich nimmer beten,<br>Und das frißt mir ins Herz hinein;<br>Dir, Heinrich, muß es auch so sein."<br>"Du hast nun die Antipathie!"	25	Garten.3.5

context marked in yellow

excerpt	stanza_nr	stanza_content	scene_nr	scene
mg2	66	"Ich muß nun fort." "Ach kann ich nie<br>Ein Stündchen ruhig dir am Busen hängen<br>Und Brust an Brust und Seel in Seele drängen?"<br>"Ach wenn ich nur alleine schlief!<br>Ich ließ dir gern heut nacht den Riegel offen;<br>Doch meine Mutter schläft nicht tief,<br>Und würden wir von ihr betroffen,<br>Ich wär gleich auf der Stelle tot!"<br>"Du Engel, das hat keine Not.<br>Hier ist ein Fläschchen! Drei Tropfen nur<br>In ihren Trank umhüllen<br>Mit tiefem Schlaf gefällig die Natur."	25	Garten.3.5
mg2	67	"Was tu ich nicht um deinetwillen?<br>Es wird ihr hoffentlich nicht schaden!"<br>"Würd ich sonst, Liebchen, dir es raten?"<br>"Seh ich dich, bester Mann, nur an,<br>Weiß nicht, was mich nach deinem Willen treibt,<br>Ich habe schon so viel für dich getan,<br>Daß mir zu tun fast nichts mehr übrigbleibt."	25	Garten.3.5

context marked in yellow

excerpt	stanza_nr	stanza_content	scene_nr	scene
mg2	68	*Gretchen geht.*<br>*Mephistopheles tritt auf, spricht Faust an.*<br><br>"Der Grasaff! ist er weg?" "Hast wieder spioniert?"<br>"Ich hab's ausführlich wohl vernommen,<br>Herr Doktor wurden da katechisiert;<br>Hoff, es soll Ihnen wohl bekommen.<br>Die Mädels sind doch sehr interessiert,<br>Ob einer fromm und schlicht nach altem Brauch.<br>Sie denken: duckt er da, folgt er uns eben auch."	26	Garten.3.6
mg2	69	"Du Ungeheuer siehst nicht ein,<br>Wie diese treue liebe Seele<br>Von ihrem Glauben voll,<br>Der ganz allein<br>Ihr seligmachend ist, sich heilig quäle,<br>Daß sie den liebsten Mann verloren halten soll."	26	Garten.3.6
mg2	70	"Du übersinnlicher sinnlicher Freier,<br>Ein Mägdelein nasführet dich."<br>"Du Spottgeburt von Dreck und Feuer!"<br>"Und die Physiognomie versteht sie meisterlich:<br>In meiner Gegenwart wird's ihr, sie weiß nicht wie,<br>Mein Mäskchen da weissagt verborgnen Sinn;<br>Sie fühlt, daß ich ganz sicher ein Genie,<br>Vielleicht wohl gar der Teufel bin.<br>Nun, heute nacht -?" "Was geht dich's an?"<br>"Hab ich doch meine Freude dran!"	26	Garten.3.6

context marked in yellow
