# Supplementary material for: Predicting subjective ratings of affect and comprehensibility with text features: a reader response study of narrative poetry
Source: Front Psychol. 2024 Oct 8;15:1431764. doi: 10.3389/fpsyg.2024.1431764 (PMC11494826; doi:10.3389/fpsyg.2024.1431764)
Supplement: Supplementary file 2 [file Table_2.pdf]

Appendix 2: Text properties used to predict subjective ratings of affect and comprehensibility

text property	full name	computation	remarks
aro_m	mean Arousal potential	word Arousal potential base values were looked up for each content word* in GLEAN (Lüdtke & Hugentobler, 2022): https://osf.io/a6w53/	a measure that quantifies the approximate evocativeness for emotional excitability of the textual context that a word is statistically likely to be found in, based on vector space modelling in the SdeWaC database: https://www.ims.uni-stuttgart.de/ forschung/ ressourcen/korpora/ sdewac/ <br>Arousal as a term for emotional excitability is inspired by the two-dimensional model of emotional intensity (Bradley & Lang, 1999)
aro_shift	Arousal shift	difference between the mean of the current and the previous Arousal potential page means (Jacobs, 2023), starting with zero before the first page <br>word Arousal base values were looked up for each content word* in GLEAN (Lüdtke & Hugentobler, 2022): https://osf.io/a6w53/	shifts between the mean emotional potentials of pages may give an insight into the dynamics of the emotional arc of a narrative (Reagan et al., 2016)
aap_m	mean Aesthetic-affective potential (AAP)	word AAP base values were looked up for each content word* in the 120k-words SentiArt database of the German language (Jacobs, 2019): https://github.com/matinho13/SentiArt	a measure that quantifies combined aspects of emotional valence and stylistic beauty of the textual context that a word is statistically likely to be found in, based on vector space modelling in the SdeWaC database:

Footnote (below the table): **content words were defined as one of the treetagger (https://www.cis.uni-muenchen.de/~schmid/tools/TreeTagger/) category tags identifying verbs, nouns, adjectives and adverbs, i.e.: ADJA, ADJD, ADV, NN, NE, VVFIN, VVIMP, VVINF, VVIZU, VVPP, VAFIN, VAIMP, VAINF, VAPP, VMFIN, VMINF, VMPP*

			https://www.ims.uni-stuttgart.de/ forschung/ ressourcen/korpora/sdewac/<br><br>Valence as a term for emotional positivity/negativity is inspired by the two-dimensional model of emotional intensity (Bradley & Lang, 1999)<br><br>AAP may be related to subjective liking (Jacobs & Kinder, 2019)
aap_pnr	positive-negative ratio of AAP	number of content words with an associated AAP-value > 0, divided by the number of content words with an associated AAP-value < 0 (Jacobs, 2023)	
joy_m	mean Joy potential	word base values were looked up for each content word* in the 120k-words SentiArt database of the German language (Jacobs, 2017): https://github.com/matinho13/SentiArt	the 5 basic emotions joy, sadness, fear, anger, and disgust (Westbury et al., 2015), specifically Anger and Disgust have been related to Arousal (Jacobs & Kinder, 2021)
sad_m	mean Sadness potential		
fear_m	mean Fear potential		
anger_m	mean Anger potential		
disg_m	mean Disgust potential		
imag_m	mean Imageability potential	word Imageability potential base values were looked up for each content word* in GLEAN (Lüdtke & Hugentobler, 2022): https://osf.io/a6w53/	a measure that quantifies the approximate evocativeness for mental images of the textual context that a word is statistically likely to be found in, based on vector space modelling in the SdeWaC database:

**content words were defined as one of the treetagger (https://www.cis.uni-muenchen.de/~schmid/tools/TreeTagger/) category tags identifying verbs, nouns, adjectives and adverbs, i.e.: ADJA, ADJD, ADV, NN, NE, VVFIN, VVIMP, VVINF, VVIZU, VVPP, VAFIN, VAIMP, VAINF, VAPP, VMFIN, VMINF, VMPP*

			https://www.ims.uni-stuttgart.de/ forschung/ ressourcen/korpora/ sdewac/<br><br>Imageability (Westbury et al., 2013) can be considered a stylistic device that has the goal of evoking mental simulation
concr_m	mean Concreteness potential	word Concreteness potential base values were looked up for each content word* in GLEAN (Lüdtke & Hugentobler, 2022): https://osf.io/a6w53/	a measure that quantifies the approximate evocativeness for non-abstract concepts of the textual context that a word is statistically likely to be found in, based on vector space modelling in the SdeWaC database: https://www.ims.uni-stuttgart.de/ forschung/ ressourcen/korpora/ sdewac/<br><br>Concreteness (Brysbaert, Warriner, & Kuperman, 2014) can be considered a stylistic device that has the goal of evoking a sense of familiarity and plausibility
sonority_m	mean Sonority score	word Sonority (Vennemann, 1987, as cited in Jacobs, 2017) base values were computed by adding each letter's sonority value in a word and then dividing that sum by the number of letters in that word<br><br>letter sonority values are based on a rank-based scoring system for the German language: 'a' = 7 points; 'e', 'o' = 6 points; 'i', 'u' = 5 points; 'j', 'w', 'y' = 4 points; 'l', 'r' = 3 points, 'm', 'n' = 2 points; all other letters = 1 point	attempt to quantify the pronounceability and auditory beauty of a word that have been linked to longer reading times (Xue et al., 2019)

**content words were defined as one of the treetagger (https://www.cis.uni-muenchen.de/~schmid/tools/TreeTagger/) category tags identifying verbs, nouns, adjectives and adverbs, i.e.: ADJA, ADJD, ADV, NN, NE, VVFIN, VVIMP, VVINF, VVIZU, VVPP, VAFIN, VAIMP, VAINF, VAPP, VMFIN, VMINF, VMPP*

logfreq_m	mean logarithmic frequency	the log-corrected base word frequency values were looked up for each content word* in the SUBTLEX database for the German language (Brysbaert et al., 2011): https://osf.io/py9ba/#!	the more frequent a word is in everyday language, the more accessible it generally is, contributing to a text's comprehensibility
nhfn_m	number of more highly frequent orthographic neighbours (NHFN)	we defined orthographic neighbours as words that differ from the base word in only a single letter<br>the log-corrected base word frequency values were looked up for each content word* in the SUBTLEX database for the German language (Brysbaert et al., 2011): https://osf.io/py9ba/#!	the assumption here is that the number of higher-frequency (orthographic) neighbours makes a word harder to process because of competition effects
lsi	Line-syllable index	product of the number of words multiplied with the average number of syllables in a line; syllables being calculated with the Python package *syllapy*: https://pypi.org/project/syllapy/	based on the readability measure *sentence-syllable index* (Jacobs, 2023), but applied to lines instead of sentences for our poetic text
log_ttr	logarithmic type-token ratio (TTR)	logarithmic quotient of the set of words length divided by the list of words length, i.e., create a list of all words in a text block without repeating any, and divide the number of items in that list by the number of total words (including repetitions) in that same text unit	a measure of morphological complexity / lexical diversity of a text (Kettunen, 2014)
cwr	content word ratio	quotient of the number of content words* divided by number of all words in a text unit	a measure of semantic density in a text (cf. Segalowitz & Lane, 2000)
avq	adjective-verb quotient	quotient of the number of adjectives* divided by the number of verbs* in a text unit	another measure of morphological complexity / lexical diversity of a (poetic) text (Simonton, 1989)
page_nr	page number in excerpt	count pages since start of the excerpt	an indication of the current page's position within the narrative structure

**content words were defined as one of the treetagger (https://www.cis.uni-muenchen.de/~schmid/tools/TreeTagger/) category tags identifying verbs, nouns, adjectives and adverbs, i.e.: ADJA, ADJD, ADV, NN, NE, VVFIN, VVIMP, VVINF, VVIZU, VVPP, VAFIN, VAIMP, VAINF, VAPP, VMFIN, VMINF, VMPP*

n_prob_evb	number of probable event boundaries	An independent set of subjects rated for each text line on whether they perceive a narrative event boundary in it (*N* = 10). The line-specific event probability was then defined as the percentage of subjects that rated a line as having a boundary. The number of probable event boundaries of a text page is the number of lines on a page that had a probability above 20%.	approach loosely inspired by the self-paced event structuring of the visual-continuous subject group in Zack, Speer, & Reynolds (2009)
snd_m	mean Semantic neighbourhood density	the semantic neighbour density (SND) is the semantic similarity of a content word* to all of its orthographic neighbours<br><br>we defined orthographic neighbours as words that differ from the base word in only a single letter in the SUBTLEX database for the German language (Brysbaert et al., 2011): https://osf.io/py9ba/#!<br><br>the semantic similarity was calculated with the german model of *word2vec/keyed_vectors* from *gensim.models* (https://radimrehurek.com/gensim/models/word2vec.html)	SND is known to affect word meaning retrieval (Hameau, Nickels, & Biedermann, 2019)
prev_page_sim	semantic similarity to previous page	the semantic similarity between the pages was calculated with the german model of *word2vec/keyed_vectors* from *gensim.models* (https://radimrehurek.com/gensim/models/word2vec.html)	inspired by the SSIM measure in Jacobs (2023) as a measure of semantic / stylistic cohesion throughout a text unit
eigensim	Semantic eigensimilarity	a page's semantic similarity to all other pages in the excerpt, calculated with the german model of *word2vec/keyed_vectors* from *gensim.models* (https://radimrehurek.com/gensim/models/word2vec.html)	inspired by a line's eigensimilarity in Jacobs (2023) as a measure of representability for the entire text (like an eigenvector in physics)

**content words were defined as one of the treetagger (https://www.cis.uni-muenchen.de/~schmid/tools/TreeTagger/) category tags identifying verbs, nouns, adjectives and adverbs, i.e.: ADJA, ADJD, ADV, NN, NE, VVFIN, VVIMP, VVINF, VVIZU, VVPP, VAFIN, VAIMP, VAINF, VAPP, VMFIN, VMINF, VMPP*

faustian_pred	Faustian predictability	the semantic predictability of the next line, within the context of the stylistics of Goethe's *Faust*<br><br>Using a version of *GPT-2* that was fine-tuned on the very work that we also extracted our stimulus material from (https://huggingface.co/dbmdz/german-gpt2-faust), the next line based on the current page was automatically generated 100 times. Then, the semantic similarity of these 100 artificial lines with the actual next line was calculated with the german model of *word2vec/keyed_vectors* from *gensim.models* (https://radimrehurek.com/gensim/models/word2vec.html) and taken as the *Faustian Predictability*.	exploratory text property
topic_01	total contribution to semantic topic 1	a page's contribution to a semantic topic of the entire text<br><br>Semantic topics were identified with a page-wise topic-modelling analysis of the entire text with MALLET (McCallum, 2002): http://mallet.cs.umass.edu. Each page's contribution to each topic was taken over as the text property describing the page's loading for the respective topic.<br><br>topic analysis seems to be a useful addition to sentiment analysis in the analysis of reader responses to literature (Jacobs, 2023; Suhendra et al., 2022)	key words: *Glaube, Ebenbild, Grab, Leben, Seele, Freude, Hölle, Erscheinung, Schelm, weissagen*
topic_02	total contribution to semantic topic 2		key words: *Schauspiel, scheinen, Tätigkeit, Wurm, Tod, Ungeheuer, Seele, Meister, schweben, Kopf*
topic_03	total contribution to semantic topic 3		key words: *Herz, Welt, Zeit, Mensch, Herr, Brust, Gott, Tag, Erde, Leben, Sinn*
topic_04	total contribution to semantic topic 4		key words: *Lust, Mutter, Auge, Vater, Pfarrer, Schau, erkennen, Nase, Tropfen, Tür*
topic_05	total contribution to semantic topic 5		key words: *Feuer, Wasser, Mann, Buch, Jammer, Freier, Bächlein, erklären, bringen, halten*

**content words were defined as one of the treetagger (https://www.cis.uni-muenchen.de/~schmid/tools/TreeTagger/) category tags identifying verbs, nouns, adjectives and adverbs, i.e.: ADJA, ADJD, ADV, NN, NE, VVFIN, VVIMP, VVINF, VVIZU, VVPP, VAFIN, VAIMP, VAINF, VAPP, VMFIN, VMINF, VMPP*

References

Bradley, M. M., & Lang, P. J. (1994). Measuring emotion: The self-assessment manikin and the semantic differential. *Journal of Behavior Therapy and Experimental Psychiatry*, *25*(1), 49–59. https://doi.org/10.1016/0005-7916(94)90063-9

Brysbaert, M., Buchmeier, M., Conrad, M., Jacobs, A. M., Bölte, J., and Böhl, A. (2011). The word frequency effect: a review of recent developments and implications for the choice of frequency estimates in German. *Exp. Psychol. 58*, 412–424. doi: 10.1027/1618-3169/a000123

Brysbaert, M., Warriner, A. B., and Kuperman, V. (2014). Concreteness ratings for 40 thousand generally known English word lemmas. *Behav. Res. 46*, 904–911. doi: 10.3758/s13428-013-0403-5

Hameau, S., Nickels, L., & Biedermann, B. (2019). Effects of semantic neighbourhood density on spoken word production. *Quarterly Journal of Experimental Psychology*, *72*(12), 2752–2775. https://doi.org/10.1177/1747021819859850

Jacobs, A.M. (2017) Quantifying the Beauty of Words: A Neurocognitive Poetics Perspective. *Front. Hum. Neurosci. 11*:622. doi: 10.3389/fnhum.2017.00622

Jacobs, A. M. (2023). *Neurocomputational Poetics: How the Brain Processes Verbal Art*. London: Anthem Press. https://doi.org/10.2307/jj.7994683

Jacobs, A. M., & Kinder, A. (2019). Computing the Affective-Aesthetic Potential of Literary Texts. *AI*, *1*(1), 11–27. https://doi.org/10.3390/ai1010002

Jacobs, A. M., & Kinder, A. (2021). *Electoral Programs of German Parties 2021: A Computational Analysis Of Their Comprehensibility and Likeability Based On SentiArt*. https://doi.org/10.48550/ARXIV.2109.12500

Kettunen, K. (2014). Can Type-Token Ratio be Used to Show Morphological Complexity of Languages? *Journal of Quantitative Linguistics*, *21*(3), 223–245. https://doi.org/10.1080/09296174.2014.911506

Lüdtke, J. & Hugentobler, K.G. (2022). Using emotional word ratings to extrapolated norms for valence, arousal, imagebility and concreteness: The German list of extrapolated affective norms (GLEAN). In: Ferstl, E., Konieczny, L., von Stülpnagel, R., Beck, J., and Zacharski, L., (Eds.). Proceedings of KogWis2022, the 5th Biannual Conference of the German Society for Cognitive Science. Albert-Ludwigs-Universität Freiburg, Freiburg, Germany. Sep. 5th-7th 2022. doi: 10.6094/UNIFR/229611 (poster).

McCallum, A. K. (2002). MALLET: A machine learning for language toolkit. URL (last checked 26 June 2012). http://mallet.cs.umass.edu

Reagan, A. J., Mitchell, L., Kiley, D., Danforth, C. M., & Dodds, P. S. (2016). The emotional arcs of stories are dominated by six basic shapes. *EPJ Data Science*, *5*(1), 31. https://doi.org/10.1140/epjds/s13688-016-0093-1

Segalowitz, S. J., and Lane, K. C. (2000). Lexical access of function versus content words. *Brain Lang.* 75, 376–389. doi: 10.1006/brln.2000.2361

Simonton, D. K. (1989). Shakespeare's sonnets: a case of and for single-case historiometry. *J. Pers.* 57, 695–721. doi: 10.1111/j.1467-6494.1989.tb00568.x

**content words were defined as one of the treetagger (https://www.cis.uni-muenchen.de/~schmid/tools/TreeTagger/) category tags identifying verbs, nouns, adjectives and adverbs, i.e.: ADJA, ADJD, ADV, NN, NE, VVFIN, VVIMP, VVINF, VVIZU, VVPP, VAFIN, VAIMP, VAINF, VAPP, VMFIN, VMINF, VMPP*

Suhendra, N. H., Keikhosrokiani, P., Asl, M. P., & Zhao, X. (2022). Opinion Mining and Text Analytics of Literary Reader Responses: A Case Study of Reader Responses to KL Noir Volumes in Goodreads Using Sentiment Analysis and Topic. In P. Keikhosrokiani & M. Pourya Asl (Eds.), *Handbook of Research on Opinion Mining and Text Analytics on Literary Works and Social Media* (pp. 191-239). IGI Global. https://doi.org/10.4018/978-1-7998-9594-7.ch009

Vennemann, T. (1987). *Preference laws for syllable structure: And the explanation of sound change with special reference to German, Germanic, Italian, and Latin*. de Gruyter.

Westbury C. F., Shaoul C., Hollis G., Smithson L., Briesemeister B. B., Hofmann M. J., & Jacobs A. M. (2013). Now you see it, now you don't: On emotion, context, & the algorithmic prediction of human imageability judgments. *Frontiers in Psychology*, 4, 991.

Westbury, C., Keith, J., Briesemeister, B. B., Hofmann, M. J., & Jacobs, A. M. (2015). Avoid violence, rioting, and outrage; approach celebration, delight, and strength: Using large text corpora to compute valence, arousal, and the basic emotions. Quarterly Journal of Experimental Psychology, 68(8), 1599-1622. https://doi.org/10.1080/17470218.2014.970204

Xue, S., Lüdtke, J., Sylvester, T., & Jacobs, A. M. (2019). Reading shakespeare sonnets: combining quantitative narrative analysis and predictive modeling—an eye tracking study. *Journal of Eye Movement Research*, *12*(5).

Zacks, J. M., Speer, N. K., & Reynolds, J. R. (2009). Segmentation in reading and film comprehension. *Journal of Experimental Psychology: General, 138*(2), 307–327. https://doi.org/10.1037/a0015305

**content words were defined as one of the treetagger (https://www.cis.uni-muenchen.de/~schmid/tools/TreeTagger/) category tags identifying verbs, nouns, adjectives and adverbs, i.e.: ADJA, ADJD, ADV, NN, NE, VVFIN, VVIMP, VVINF, VVIZU, VVPP, VAFIN, VAIMP, VAINF, VAPP, VMFIN, VMINF, VMPP*
